# Integrated Analysis to Reveal Heterogeneity of Tumor‐Associated Neutrophils in Glioma

**DOI:** 10.1002/cam4.70745

**Published:** 2025-03-07

**Authors:** Wen Wang, Junsheng Li, Qiheng He, Chenglong Liu, Siyu Wang, Zhiyao Zheng, Bojian Zhang, Siqi Mou, Wei Sun, Jizong Zhao

**Affiliations:** ^1^ Department of Neurosurgery Beijing Tiantan Hospital, Capital Medical University Beijing China; ^2^ China National Clinical Research Center for Neurological Diseases Beijing China

**Keywords:** biomarker, glioma, introduction, prognosis, scRNA‐seq, tumor‐associated neutrophil

## Abstract

**Background:**

Glioma, characterized by its cellular and molecular heterogeneity, presents formidable challenges in treatment strategy and prognostic assessment. The tumor microenvironment (TME) profoundly influences tumor behavior and treatment response, with tumor‐associated neutrophils (TANs) playing a complex but understudied role. This study aimed to investigate the heterogeneity and role of TANs in glioma and to develop a prognostic model.

**Methods:**

Analysis of scRNA‐seq data identified cellular subpopulations and differentially expressed neutrophil‐related genes (DE‐NRGs). Bulk RNA‐seq was obtained from four independent datasets. Molecular subtypes of glioma samples were determined by consensus clustering. WGCNA was conducted to elucidate the association between gene modules and subtypes. We developed a risk score model. Expression of selected genes was confirmed using immunohistochemistry (IHC). In vitro experiments were also performed for functional verification, including CCK8, EdU, Transwell, and TUNEL assays.

**Results:**

A total of 108 DE‐NRGs for TANs were identified based on scRNA‐seq data. Two molecular subtypes were characterized, showing significant differences in prognosis and clinical features. Immune‐related analyses demonstrated varied immunological characteristics between subtypes. The risk score model was constructed with 7 genes, including AEBP1, CAVIN1, DCTD, DEPP1, DUSP6, FKBP9, and UGCG. It showed significant prognostic value and was validated across three external datasets. The mutation landscape highlighted higher IDH mutation prevalence in low‐risk groups. Drug sensitivity analysis indicated TMZ resistance in high‐risk groups. In vitro experiments showed that UGCG could promote glioma cell proliferation, migration, and invasion, while decreasing apoptosis.

**Conclusion:**

This study explored the heterogeneity of TANs and developed a prognostic model, providing insights for prognostic prediction and guiding personalized treatment strategies in glioma.

Declaration of Generative AI in Scientific Writing: The authors declare nonuse of generative AI and AI‐assisted technologies in the writing process.

## 1 Introduction

Gliomas exhibit considerable heterogeneity in cellular composition and molecular characteristics, contributing to the challenges in understanding their complex pathogenesis and pathobiology [1]. Despite the implementation of standard treatment strategies, patients often have local recurrence, malignant progression, and poor prognosis due to the invasive and infiltrative growth characteristics of glioma [2, 3]. Recent advancements in multi‐omics technology have propelled research toward identifying prognostic biomarkers [4, 5]. The World Health Organization (WHO) incorporated molecular diagnostics into the classification of CNS tumors in 2016 and further expanded on molecular characteristics in 2021, emphasizing the importance of molecular evaluation in glioma diagnosis and prognosis [6, 7]. Despite advancements in molecular biomarkers, accurately predicting glioma prognosis remains a formidable task. Further investigations are imperative to delineate molecular subtypes, promoting the discovery of innovative prognostic biomarkers and effective therapeutic targets.

Tumor microenvironment (TME) has emerged as a critical determinant of disease progression, therapeutic response, and patient prognosis [8]. Constituted by a complex interplay of diverse cell types, extracellular matrix components, and soluble factors, the TME profoundly shapes tumor behavior and therapeutic outcomes, particularly in the context of immunotherapy [9]. Within the TME, neutrophils exhibit a high degree of diversity and plasticity, playing a double‐edged role as promoters or inhibitors of tumor progression. In recent years, there has been a growing focus on unraveling the multifaceted roles of tumor‐associated neutrophils (TANs) [10]. Neutrophils have been recognized not only for their traditional role in host defense but also for their capacity to modulate tumor growth, angiogenesis, and immune evasion [11]. TANs have been found to be valuable prognostic biomarkers in various cancer types [12]. However, the understanding of TANs in glioma remains relatively limited.

In recent years, single‐cell RNA sequencing (scRNA‐seq) has revolutionized our ability to identify the heterogeneity of the tumor immune microenvironment (TIME) [13]. It enables the comprehensive profiling of transcriptomic features at the individual cell level, facilitating the identification and characterization of distinct cell types, as well as the delineation of cell states within the TIME [14]. In this study, we investigated the role of TANs in glioma, identified predictive biomarkers of immunotherapy response, and developed innovative therapeutic strategies aimed at reprogramming the glioma immune landscape. Additionally, we constructed and validated a prognostic model for accurate prediction in glioma patients.

## 2 Methods

### 2.1 Data Collection

**FIGURE 1:**
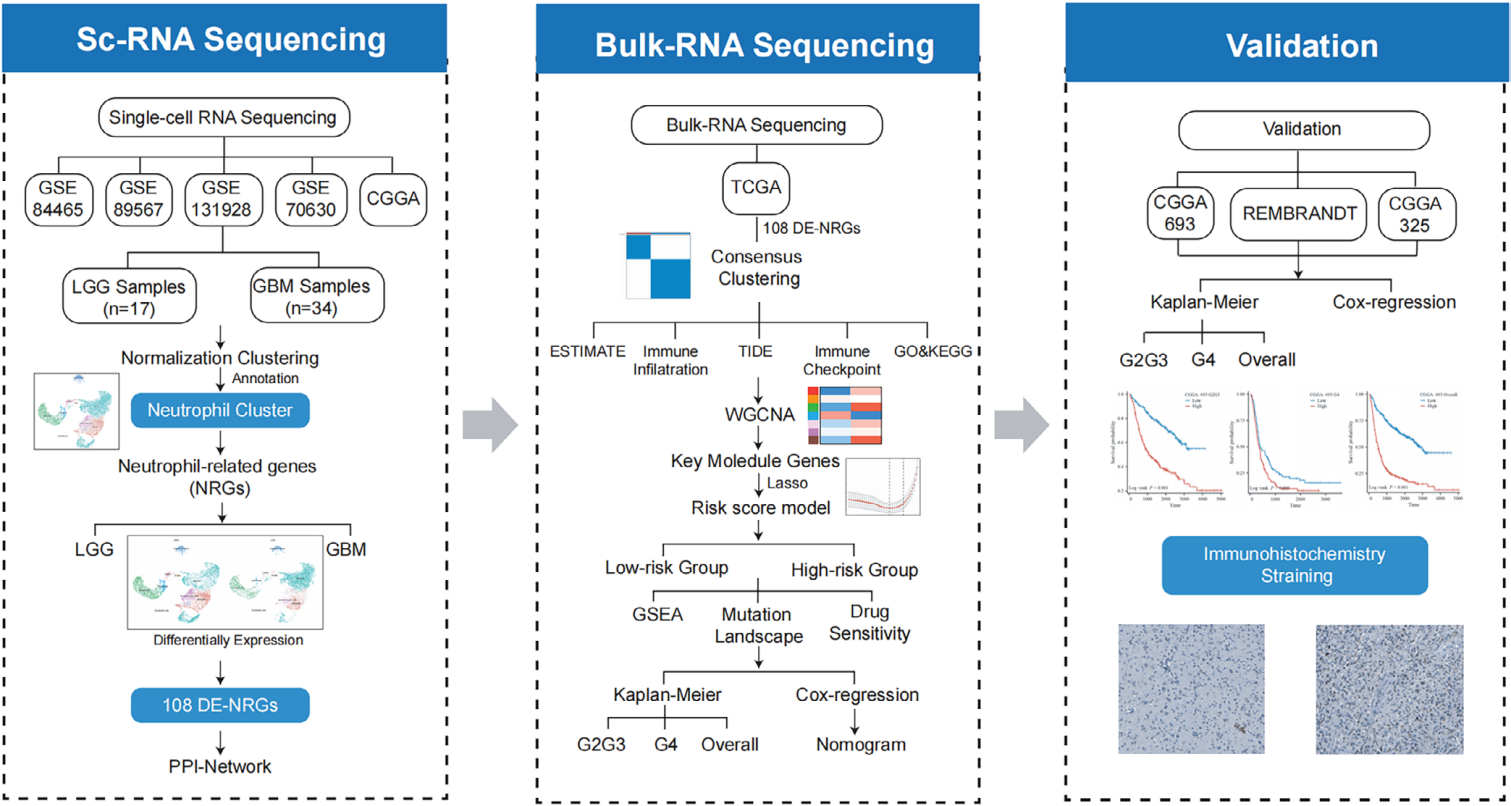
Flowchart outlining the study design.

We collected scRNA‐seq of 51 glioma samples from GSE84465, GSE89567, GSE131928, GSE70630, and CGGA datasets, comprising 17 LGG and 34 GBM samples [15, 16]. Bulk RNA‐seq of 2194 glioma samples was collected, including 701 samples from TCGA database, 693 samples from CGGA‐693 dataset, 325 samples from CGGA‐325 dataset, and 475 from REMBRANDT dataset [16, 17, 18]. The RNA‐seq and corresponding clinical information were collected from TCGA database (https://portal.gdc.cancer.gov/). Three RNA‐seq datasets (CGGA‐693, CGGA‐325, REMBRANDT) were collected from CGGA database (http://www.cgga.org.cn/). All clean data were analyzed in the study. We presented the study design in the flowchart (Figure 1).

### 2.2 Processing scRNA‐Seq Data

The quality control was conducted by filtering out genes present in fewer than 3 cells, or cells with fewer than 50 identified features, or cells containing more than 5% mitochondrial genes or more than 0.3% hemoglobin genes. Additionally, cell cycle evaluation was performed (Figure S1). Subsequently, the remaining data underwent normalization using the SCTransform method to address batch effects. Select 3000 highly variable genes and find anchor points using the SelectIntegrationFeatures method for integration. After reducing scRNA‐seq data dimension using PCA, a total of 17 principal components were employed for UMAP and t‐SNE (Figure S2). We annotated and identified the neutrophil cluster using the CellMarker database. The scRNA‐seq analysis was conducted using the Seurat package [19].

### 2.3 Identification of Differentially Expressed Neutrophil‐Related Genes

Initially, utilizing the Seurat function FindAllMarkers with specific thresholds (min‐pct set at 0.3, |logFC| > 2, and P.adj < 0.05), we computed marker genes associated with neutrophils. These identified genes, exhibiting the highest expression levels in neutrophils, were denoted as neutrophil‐related genes (NRGs). Afterward, we compared the differentially expressed genes (DEGs) within the neutrophil clusters between LGG and GBM, using the Seurat function FindMarkers [20]. Subsequently, we defined the overlap between these two sets as differentially expressed neutrophil‐related genes (DE‐NRGs), which exhibited distinct expression patterns in neutrophils from LGG compared to those from GBM.

### 2.4 Construction of Interaction Network

To elucidate the interactions among the identified DE‐NRGs, we developed a PPI network using the STRING database [21]. The establishment of the PPI network utilized a minimum interaction score threshold set at 0.4. Subsequently, the network was imported and visually represented using Cytoscape software. Furthermore, we applied the MCODE algorithm to identify the most significant clusters within the PPI network.

### 2.5 Consensus Clustering

To categorize samples into distinct clusters associated with neutrophils, we performed consensus clustering through the ConsensuClusterPlus package [22]. The subtypes of samples within the TCGA database were identified utilizing the expression data of DE‐NRGs. Subsequently, 500 bootstrapping iterations were conducted using the km method, employing Canberra as the metric distance, with each bootstrap comprising 80% of the samples [23]. The optimal number of molecular subtypes was determined by calculating the consistency matrix and consistency cumulative distribution function (CDF). The establishment of molecular subtypes involved a comprehensive exploration of cluster configurations. To visualize and distinguish NRG clusters, PCA was performed. This analytical approach aids in the differentiation and characterization of distinct neutrophil‐related subtypes within the samples.

### 2.6 Immune‐Related Analyses

The ESTIMATE algorithm was performed to compute key parameters, including Immune Score, Stromal Score, ESTIMATE Score, and Tumor Purity [24]. Subsequently, the infiltrating levels of immune cells were assessed using the ssGSEA algorithm with the GSVA package [25, 26]. Furthermore, the tumor immune dysfunction and exclusion (TIDE) website was employed to calculate the TIDE score, dysfunction score, and exclusion score [27]. Additionally, we compared the expression levels of immune checkpoints between different subtypes. These analyses provided a comprehensive understanding of the immune landscape and potential mechanisms associated with immune escape and dysfunction.

### 2.7 Functional Enrichment Analyses

Using the ClusteProfiler package, we conducted GO and KEGG enrichment analyses between clusters based on DEGs with |logFC| > 1 and Padj < 0.05 [28]. Additionally, we conducted the GSEA process with ClusteProfiler to investigate functional annotations and signaling pathways. In each analysis, we performed 1000 repetitions of gene‐set permutation. A rigorous filter condition has been established, requiring a Padj value < 0.05 and an FDR value < 0.01.

### 2.8 Weighted Gene Co‐Expression Network Analysis (WGCNA)

We performed WGCNA to elucidate the intricate relationships among genes, samples, gene modules, and phenotypes. The process involved calculating the standard deviation (SD) for each gene and selecting the top 25% for subsequent analysis [29]. Using the goodSamplesGenes method of the WGCNA package, outlier genes and samples were excluded [30]. To construct a scale‐free co‐expression gene network, we determined the optimal soft‐thresholding power (β). This facilitated the formation of an adjacency matrix, which was then transformed into a topological overlap matrix (TOM), integrating similarity and dissimilarity measures. Hierarchical clustering was employed to reveal module eigengenes, providing a comprehensive summary of the identified modules. Pearson correlation was used to validate the correlation between modules and subtypes, focusing on the key modules with the most significant positive and negative correlations.

### 2.9 Development of NRG‐Related Prognostic Model

We identified key module genes with prognostic significance. Subsequently, these prognostic key module genes were incorporated into LASSO regression to construct the risk score model. By calculating the risk scores of samples, we separated all samples into different risk groups based on the median risk score.

### 2.10 Mutation Landscape and Drug Sensitivity Analysis

We identified the top 10 mutated genes of LGGs and GBMs in the TCGA database, respectively. Samples in the LGG and GBM datasets were divided into different risk groups based on the median risk scores corresponding to the two datasets. Subsequently, we compared the mutation frequencies of these genes between different risk groups. Based on the Genomics of Drug Sensitivity in Cancer (GDSC) database, we assessed the drug sensitivity of each sample using the OncoPredict package to investigate the response of samples to chemotherapeutic agents [31].

### 2.11 Survival Analysis and Nomogram Construction

We performed Kaplan–Meier analyses to evaluate the disparity in survival distribution between different risk groups within glioma samples. Patients with complete clinical data were selected for univariate and multivariate Cox regression, confirming the significance of the NRG‐related risk score in predicting prognosis. Subsequently, the same clinical characteristics as in Cox regression were integrated to develop a nomogram model using the RMS package and the survival package. The nomogram model aimed to predict OS rates at 1‐year, 3‐year, and 5‐year. Time‐dependent ROC curves and calibration plots were further generated. Furthermore, we validated the prognostic value of the NRG‐related risk score in gliomas using three external datasets, ensuring the robustness and generalizability of our findings across diverse datasets.

### 2.12 Immunohistochemistry (IHC) Staining

We obtained IHC results from the HPA database to confirm the expression of genes within the NRG‐related risk model across different grades of glioma. This validation process confirmed the alignment between protein levels and gene expression.

### 2.13 Quantitative Real‐Time Polymerase Chain Reaction (qRT‐PCR)

Total RNA was extracted using Trizol reagent (15,596,026, Thermo Fisher), and reverse‐transcribed using FastKing gDNA Dispelling RT SuperMix (KR118‐02, TIANGEN) according to the manufacturer's protocol. The qRT‐PCR was performed to detect the expression level of UGCG using the standard protocol from the SuperReal PreMix Plus (FP205‐02, TIANGEN). Glyceraldehyde‐3‐phosphate dehydrogenase (GAPDH) was used as the internal standard. The primers have been shown (Table S1).

### 2.14 Western Blot Assay

Protein samples were added to (RIPA) lysis buffer (P0013B, Beyotime) mixed with Protease Inhibitor Cocktail (CW2200, CWBIO) and Phosphatase Inhibitor Cocktail (CW2383, CWBIO), then collected using a radioimmunoprecipitation assay. Pierce BCA Protein Assay Kit (23,227, Thermo Scientific) was used to quantify the samples. Equivalent amounts of protein were separated by electrophoresis and transferred onto PVDF membranes. Membranes were blocked with Bovine Serum Albumin (A6020, Biotopped) for 1 h at room temperature and then incubated with primary antibodies overnight at 4°C. Membranes were blocked with UGCG antibody for 1 h.

### 2.15 Cell Proliferation, Migration, and Invasion Assays

The assays were performed on U87 and LN229 cell lines. For the CCK8 assay, cells were plated in 96‐well plates at a density of 2000 cells per well. After attachment, the medium in each well was replaced with 100 μL of complete medium containing 10 μL of CCK8 reagent (CA1210‐500 T, Solarbio). Cells were evaluated by measuring the absorbance using a microplate reader at 450 nm. For the EdU assay, appropriate numbers of co‐cultured tumor cells were placed in 6‐well plates. Then, add an appropriate amount of EdU (C0078S, Beyotime) according to the instructions, and complete labeling after 2 h. Cells were fixed and further stained according to the manufacturer's protocol. For the colony formation assay, cells were transfected for 24 h, and 1000 cells were plated in each well of the 6‐well plate to form a colony. The cell migration assay was performed using Transwell chambers (8 μm, Corning). Approximately 4 × 10^4^ cells were seeded into the upper chamber in serum‐free medium (200 μL) and the lower chamber added medium (500 μL) containing 20% FBS. After incubation for 12 h, cells migrating to the lower surface of the membrane were fixed and stained with 0.1% crystal violet. For the invasion assay, Matrigel Invasion Chambers in the 24‐well plates were used. Approximately 4 × 10^4^ cells were seeded into the upper chamber, and the lower chamber added medium (500 μL). Cells were fixed and stained after 24 h.

### 2.16 TUNEL Assay

U87 and LN229 cells were cultured to a suitable density and fixed with 4% paraformaldehyde for 30 min. Cells were then treated with PBS containing 0.3% Triton X‐100 at room temperature for 5 min. Add 50 μL TUNEL detection solution (C10990, Beyotime) to samples and incubate at 37°C in the dark for 60 min. Cells were observed under a fluorescence microscope (DM3000, Leica) after being washed with PBS.

### 2.17 Statistical Analysis

In this study, statistical analyses were conducted using the R project. Comparisons of continuous variables between two groups were performed using the Wilcoxon rank‐sum test, while comparisons among three or more groups were carried out using the Kruskal–Wallis test. Unordered categorical variables were analyzed with the Pearson chi‐square test, whereas ordered categorical variables were assessed using the Kruskal–Wallis test. Spearman correlation analysis was employed to evaluate associations between variables. A two‐sided *p* value of less than 0.05 was considered statistically significant for all analyses.

## 3 Results

### 3.1 scRNA‐Seq Analysis of Glioma

**FIGURE 2:**
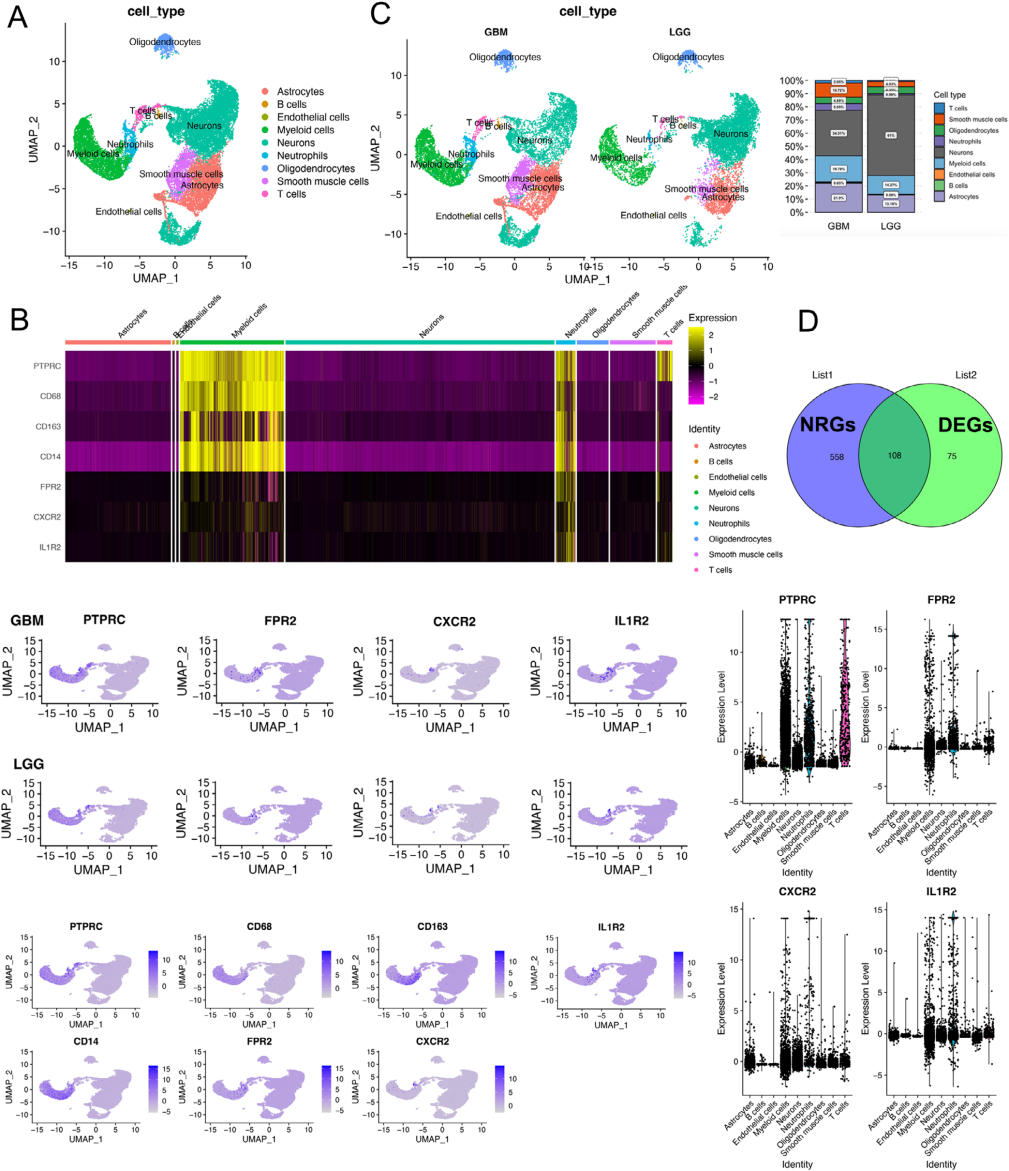
Identification of neutrophil clusters and DE‐NRGs based on scRNA‐seq data. (A) Cell clusters visualized in UMAP plots. (B) Expression of marker genes for neutrophils. (C) Variation in the proportion of cell clusters between LGG and GBM. (D) Identification of DE‐NRGs between LGG and GBM. List 1 indicates NRGs, while List 2 shows DEGs in neutrophils between LGG and GBM.

A total of 24,530 high‐quality cells from 51 glioma samples were included, comprising 10,657 cells from LGG and 13,873 cells from GBM. These cells were then separated into 9 clusters, visualized using UMAP (Figure 2A). Utilizing the CellMarker database, the cluster exhibited significantly elevated expression levels of FPR2, CXCR2, and IL1R2, indicative of neutrophils (Figure 2B). The quantity and proportion of neutrophils were assessed between LGG and GBM (Figure 2C), revealing a higher proportion in GBM (5.05%) compared to LGG (0.99%). Furthermore, a total of 108 DE‐NRGs had been identified (Figure 2D). We analyzed the PPI network of these genes and identified 2 significant modules in this network (Figure S3).

### 3.2 Identification of Molecular Subtypes and Clinical Characteristics

**FIGURE 3:**
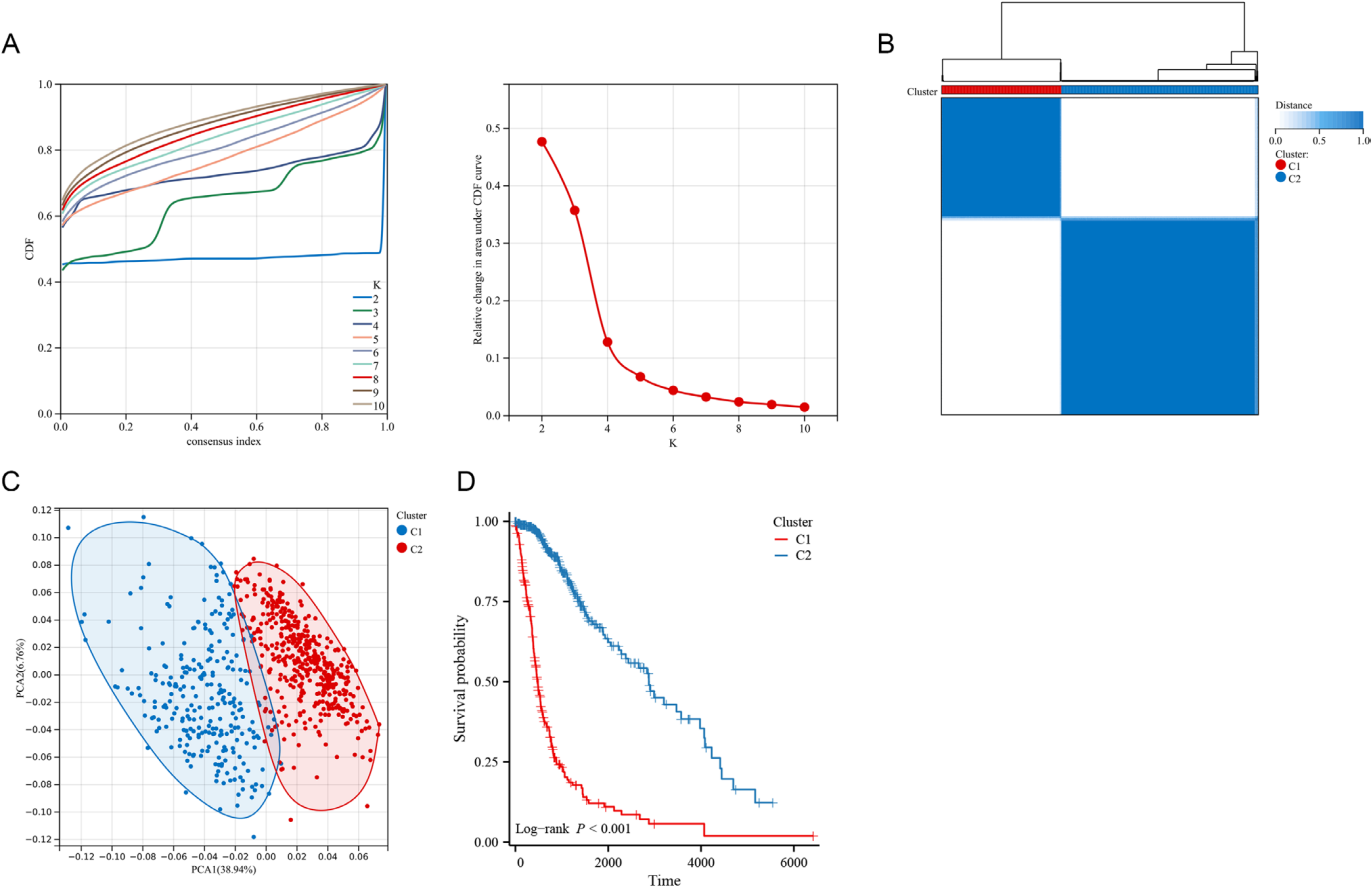
Consensus clustering of gliomas based on 108 DE‐NRGs. (A) CDF curves for consensus scores and CDF Delta area curves. (B) Consensus clustering matrix displaying two subtypes. (C) Distinction between two subtypes by PCA. (D) Kaplan–Meier curves for two subtypes.

Subsequent to the identification of 108 DE‐NRGs, the construction of molecular subtypes was undertaken. The CDF analysis indicated that selecting 2 clusters resulted in relatively stable clustering results (Figure 3A). Consequently, samples from the TCGA database were categorized into two distinct molecular subtypes (Figure 3B). Furthermore, the PCA scatter plot depicted the distribution of these two subtypes (Figure 3C), suggesting significant heterogeneity among glioma patients, possibly attributed to distinct neutrophil characteristics. Notably, these two subtypes exhibited substantial differences in prognosis (Figure 3D). Patients in the C1 subtype experienced significantly worse outcomes. Additionally, our findings indicated that samples within the C1 subtype were notably associated with older age and a significantly higher prevalence of WHO G4, IDH wild‐type, and 1p19q non‐codeletion (Figure S4).

### 3.3 Immune‐Related Analyses Between Molecular Subtypes

**FIGURE 4:**
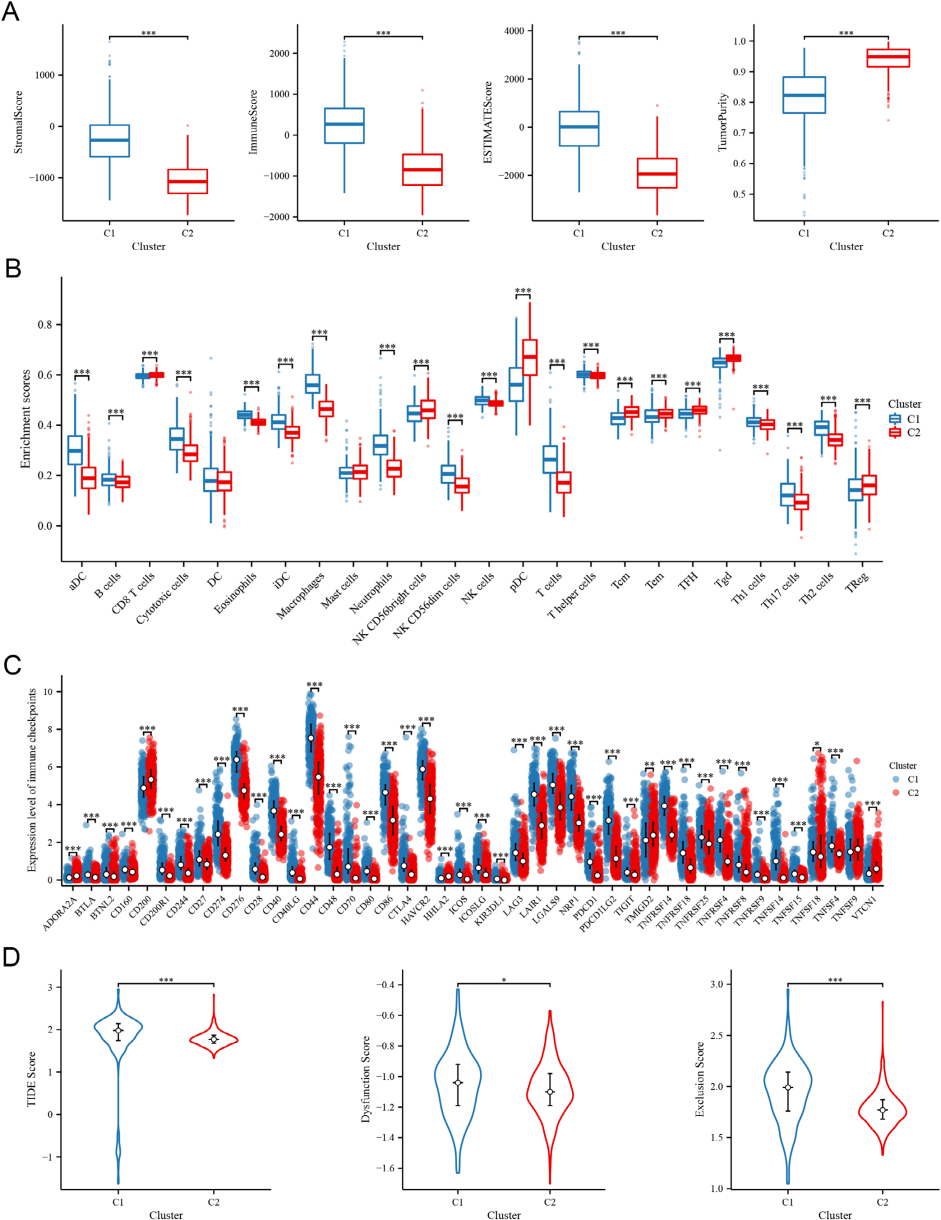
Immune‐related characteristics in two subtypes. (A) Comparison of stromal score, immune score, ESTIMATE score, and tumor purity between two subtypes. (B) Infiltration levels of immune cells between two subtypes. (C) Expression levels of immune checkpoints between two subtypes. (D) TIDE score, dysfunction score, and exclusion score between two subtypes.

The ESTIMATE analysis showed that samples of C1 subtype, which were associated with worse prognoses, exhibited significantly higher levels of three scores compared to C2 subtype. Additionally, the samples in C2 subtype showed a significantly higher tumor purity (Figure 4A, *p* < 0.05 for all). The immune infiltration analysis was performed to explore differences in the immune microenvironment between the two clusters. It showed that most immune cells were significantly increased in C1 subtype (Figure 4B). Moreover, we compared the expression of immune checkpoints, and the majority showed higher expression levels in C1 (Figure 4C). The significantly higher levels of TIDE score, dysfunction score, and exclusion score in C1 subtype indicated a higher likelihood of immune escape and less benefit from immunotherapy (Figure 4D).

### 3.4 Functional Enrichment Analyses

To discern potential differences in biological functions, we conducted GO and KEGG analyses between two molecular subtypes. It showed enrichment of genes primarily associated with biological processes related to neutrophil and immune function, thereby reinforcing the accuracy of subtype classification (Figure S5). KEGG analyses showed enrichment of pathways associated with immune response, which completed the GO enrichment results. This further underscored the association of these genes with neutrophil and immune‐related biological processes or functions.

### 3.5 Construction of NRG‐Related Risk Score Model

**FIGURE 5:**
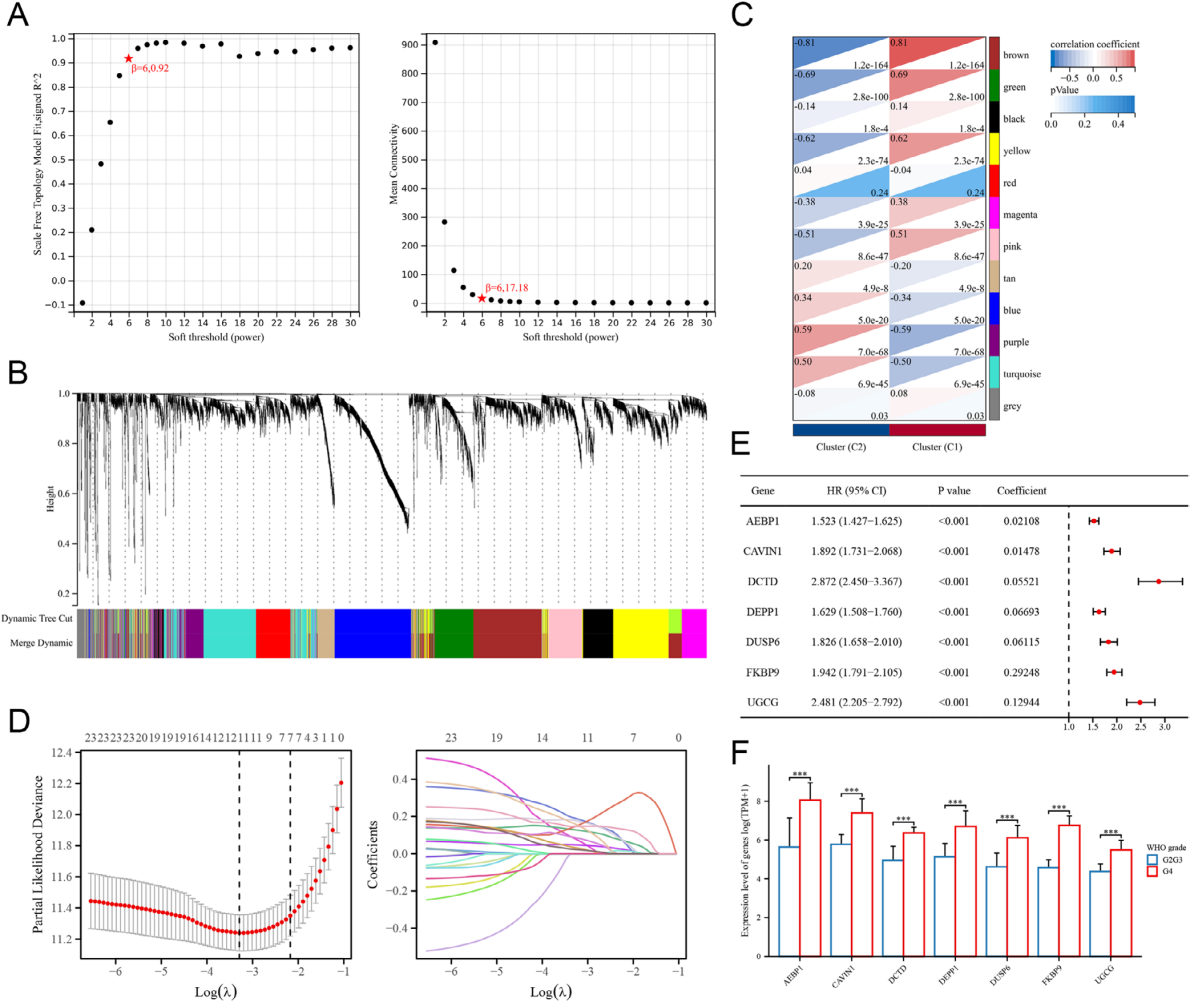
Identification of key modules by WGCNA and construction of the risk score model. (A) Scale independence and mean connectivity analysis for soft threshold power. (B) Cluster dendrogram of the coexpression network modules. (C) Module‐trait association between two subtypes. (D) Cross‐validation and coefficient profiles of LASSO regression. (E) Coefficients of the selected genes for the risk score model. (F) Expression levels of selected genes in gliomas of different grades.

Within WGCNA analysis, the optimal soft threshold was determined as 6 (Figure 5A). Subsequently, 12 co‐expressed gene modules were identified by merging modules with minModuleSize set to 100 and distances less than 0.25 (Figure 5B). It was observed that the brown module and purple module exhibited the most positive association with the NRG‐related glioma subtypes by correlation analysis (Figure 5C). The key module genes exhibiting prognostic value were further incorporated into LASSO regression. Seven genes were selected to construct the NRG‐related risk score system, including AEBP1, CAVIN1, DCTD, DEPP1, DUSP6, FKBP9, and UGCG (Figure 5D,E). The expression levels of these genes displayed a significant increase with the grade of glioma (Figure 5F).

### 3.6 Association Between Risk Score and Clinical Features

We observed a significant increase in the rates of elderly individuals, WHO G4, IDH wild‐type, and 1p19q non‐codeletion in the high‐risk group (*p* < 0.05 for all, Table S2). We further compared the levels of risk scores in different clinical subgroups. The results demonstrated a statistically significant elevation in risk scores among the elderly, IDH mutant, 1p/19q non‐codeletion groups, and C1 subtype. Furthermore, a notable trend was observed, indicating a significant increase in risk scores with the escalation of glioma grades (Figure S6).

### 3.7 GSEA and Immune Infiltration Analysis

GSEA analysis was conducted to reveal potential variances in biological functions between different risk groups. The findings indicated significant enrichment in immune response and signaling pathways associated with immunity, including epithelial–mesenchymal transition, inflammatory response, TNFα signaling via NFκB, complement, IL‐6 JAK STAT3 signaling, and IL‐2 STAT5 signaling (Figure S7). Subsequently, we compared the immune infiltrating levels between different risk groups, revealing a significant increase in the majority of immune cells within the high‐risk group. The risk score was significantly positively associated with the levels of macrophages, eosinophils, and neutrophils (Figure S8).

### 3.8 Mutation Landscape and Drug Sensitivity

We identified the top 10 mutant genes in LGG and GBM, respectively, and compared their prevalence between different risk groups. It showed a statistically significant elevation in the mutation prevalence of EGFR and PTEN in the high‐risk group of LGGs (*p* < 0.05 for both). Additionally, the frequency of ATRX mutation exhibited a significant increase in the low‐risk group of GBMs (*p* < 0.05). Notably, the frequency of IDH mutation was significantly higher in the low‐risk group of both LGGs and GBMs (*p* < 0.05 for both, Figure S9). Moreover, we evaluated the sensitivity of common chemotherapeutic drugs between different risk groups. The results indicated that the IC50 values of Temozolomide (TMZ) and Vincristine were markedly lower in the low‐risk group. Conversely, the IC50 values of other chemotherapeutic agents were significantly lower in the high‐risk group (*p* < 0.05 for all, Figure S10).

### 3.9 Prognostic Value of the NRG‐Related Risk Score

**FIGURE 6:**
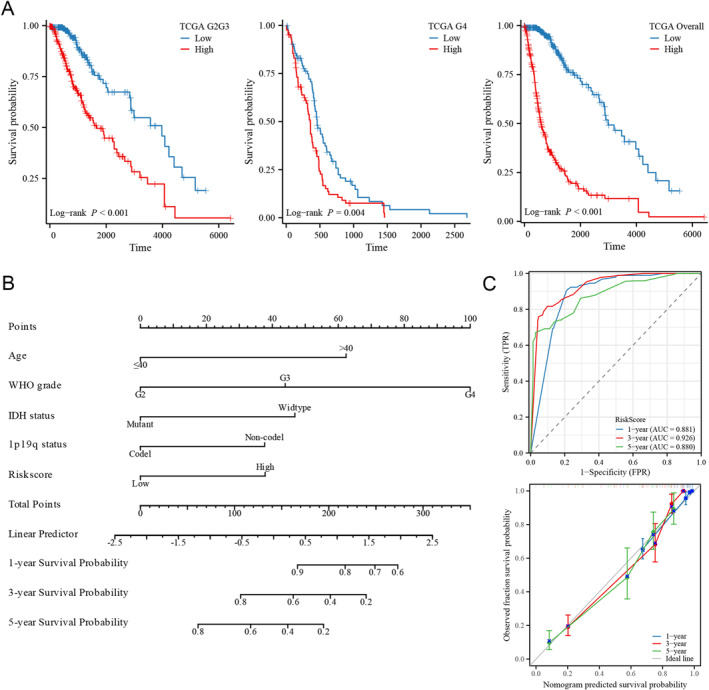
Prognostic value of the risk score in glioma patients. (A) Survival analysis in different grades of glioma in the TCGA database. (B) Predictive nomogram for glioma patients. (C) Time‐dependent ROC curves and calibration plots.

**FIGURE 7:**
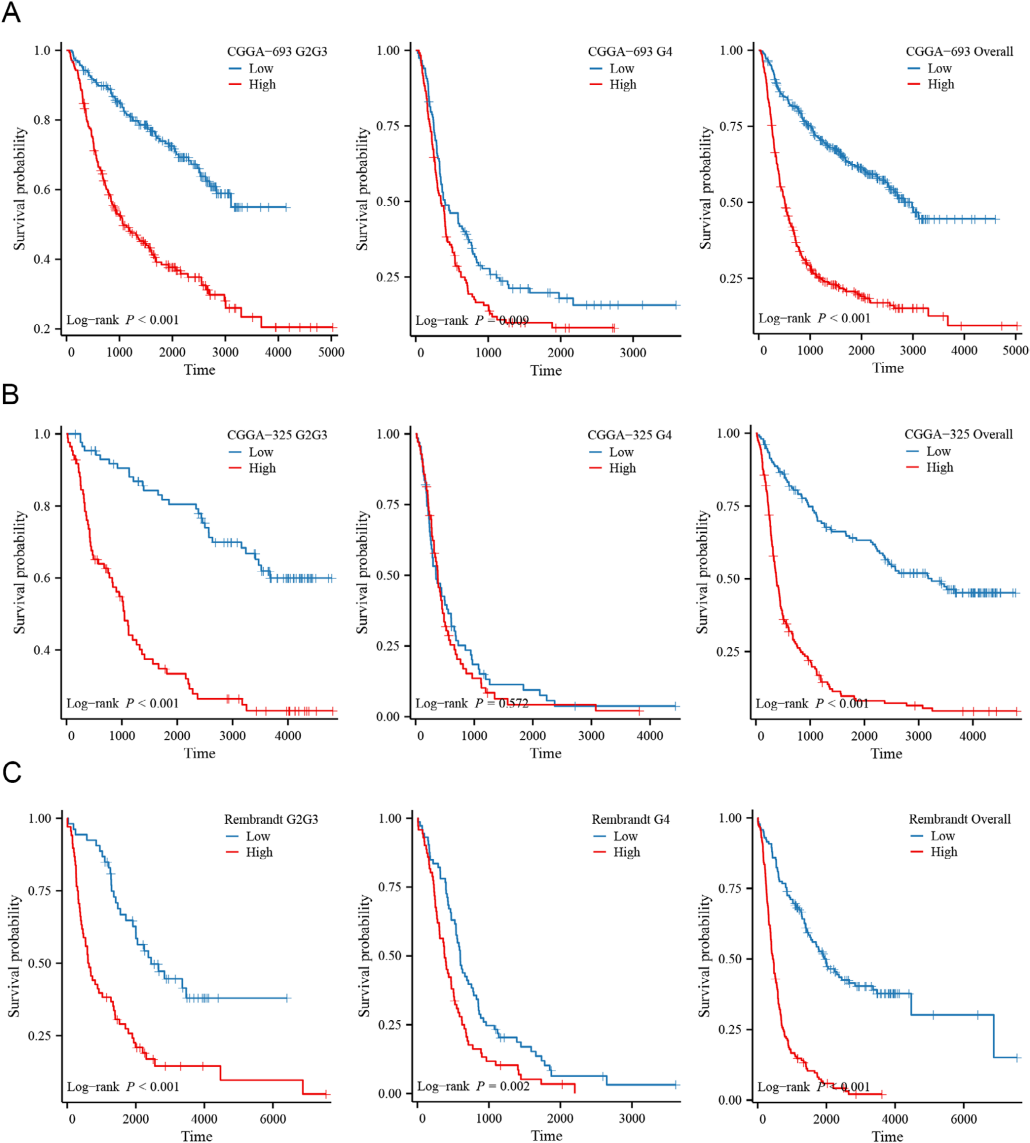
Validation of the prognostic value of the risk score in three external datasets. (A) Survival analysis in CGGA‐693 dataset. (B) Survival analysis in CGGA‐325 dataset. (C) Survival analysis in REMBRANDT dataset.

We conducted a comparison of survival distributions between different risk groups, revealing a significantly poor outcome for the high‐risk group in LGGs, GBMs, and glioma overall (*p* < 0.05 for all, Figure 6A). Cox regression identified the NRG‐related risk score as an independent prognostic factor for glioma patients (HR = 1.753, 95% CI = 1.121–2.742, *p* = 0.014, Table S3). Subsequently, the prognostic features confirmed by Cox regression were utilized for nomogram construction (Figure 6B). The concordance index of the nomogram was calculated as 0.846 (95% CI = 0.836–0.856). Time‐dependent ROC curves illustrated AUC values of 0.881, 0.926, and 0.880 for 1, 3, and 5 years, respectively. Calibration plots further supported the favorable predictive accuracy of the nomogram (Figure 6C). Additionally, we validated the prognostic value in three external datasets, including CGGA‐693, CGGA‐325, and REMBRANDT cohorts. Kaplan–Meier curves demonstrated that glioma patients in the high‐risk group exhibited a shorter OS compared to those in the low‐risk group across different grades, including WHO G2G3, G4, and glioma overall, although variations in risk scores in the G4 of the CGGA‐325 dataset had a minimal effect on the survival of patients (Figure 7). Consistent with the results of Cox regression in the TCGA dataset, the risk score was independently validated as a prognostic factor for glioma patients in all three external datasets (Tables S4–S6).

### 3.10 Role of UGCG in Glioma

**FIGURE 8:**
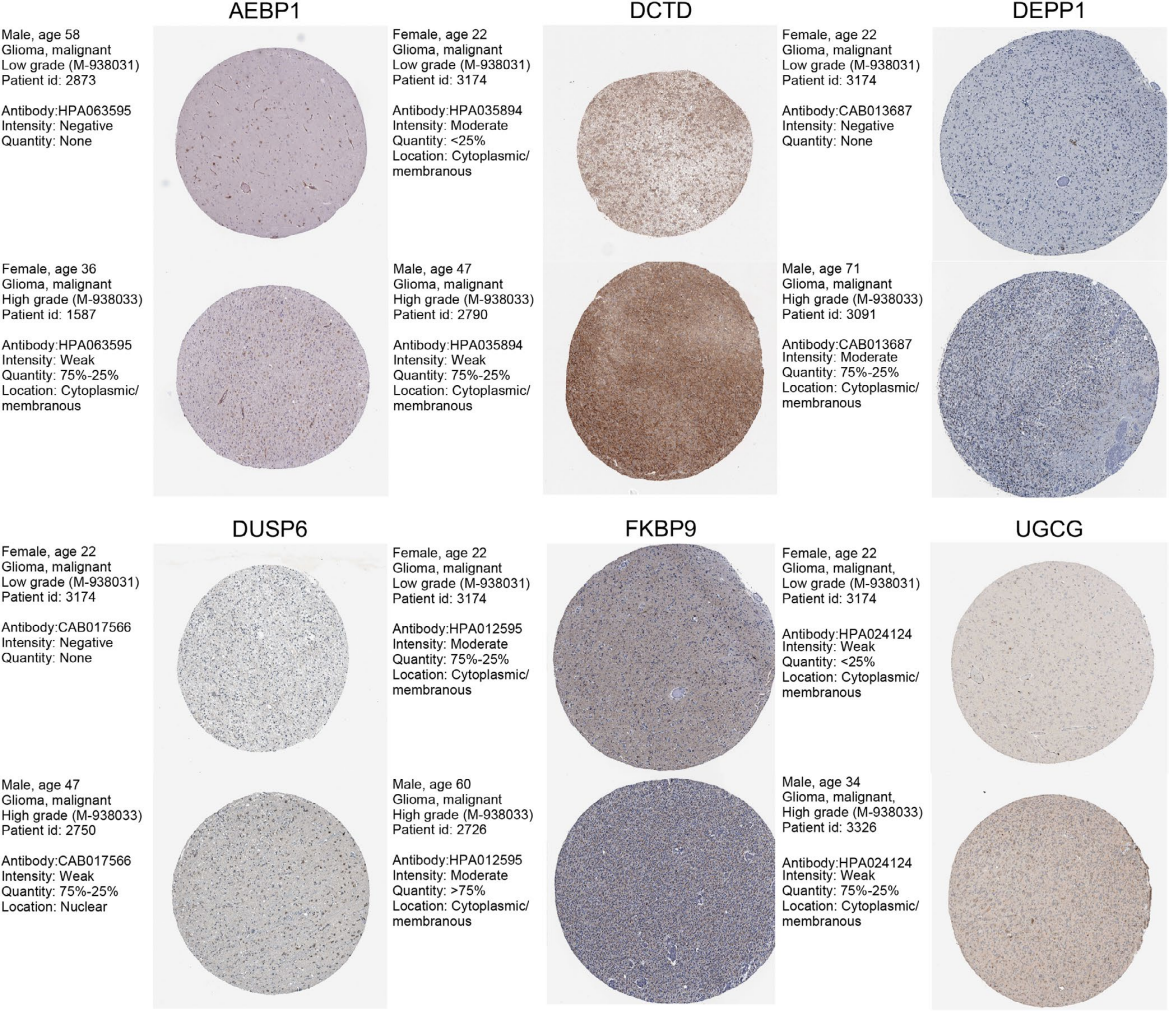
IHC staining validating the expression of the selected genes in glioma samples from the HPA database.

**FIGURE 9:**
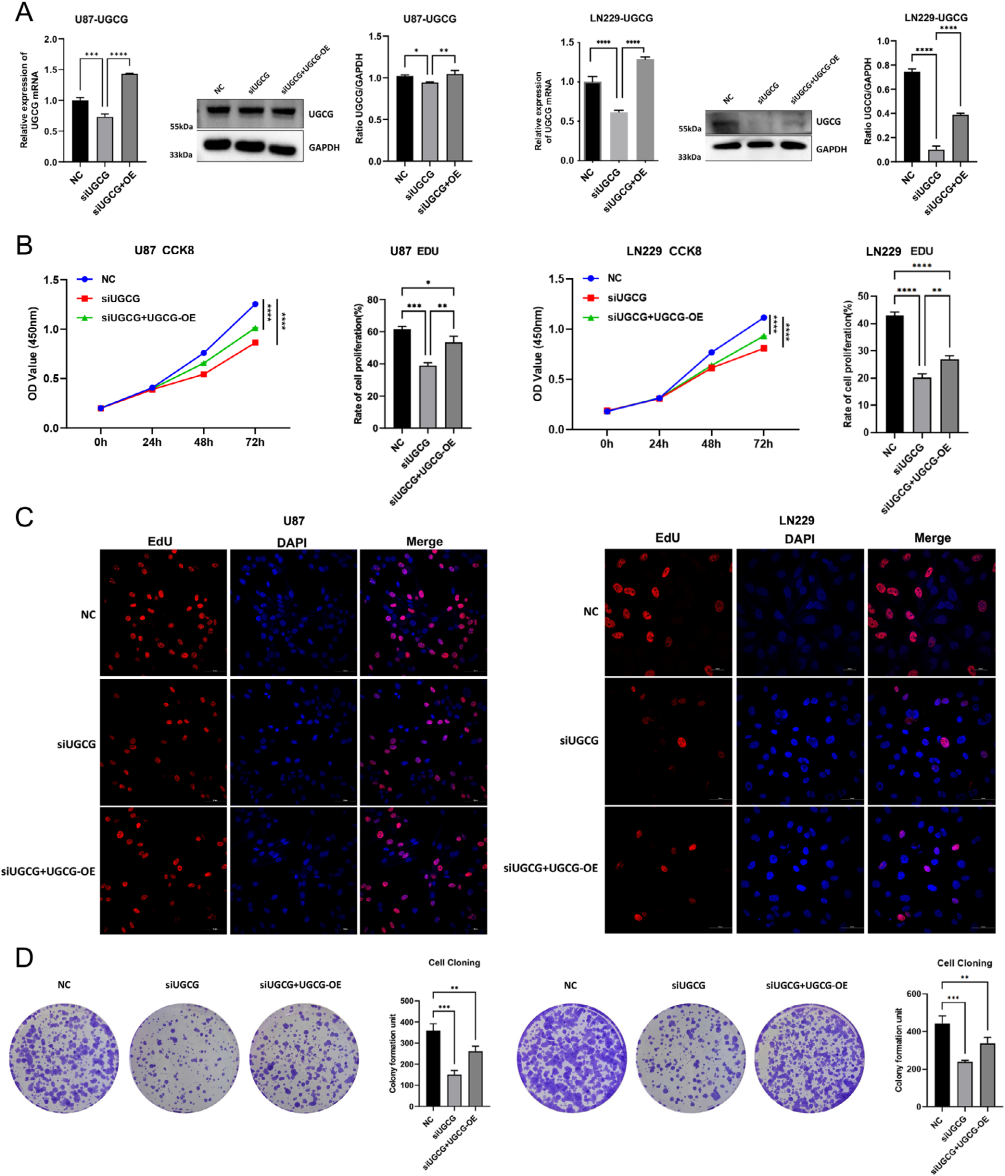
UGCG promoted cell proliferation in vitro. (A) The expression of UGCG at both transcriptomic and protein levels in U87 and LN229. (B and C) Line graph showing CCK‐8 assays at 24, 48, and 72 h. Representative image of Edu assay in U87 and LN229. (D) Colony formation assay was conducted to evaluate the effect of UGCG on cloning ability in U87 and LN229.

**FIGURE 10:**
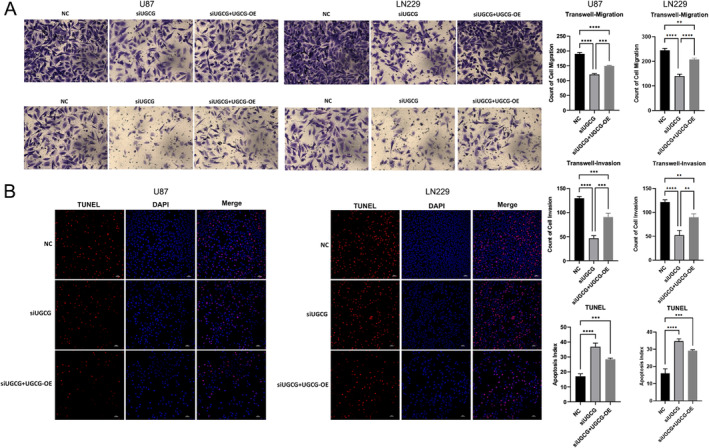
UGCG promoted cell migration and invasion, while decreasing apoptosis of glioma in vitro. (A) Representative image of transwell migration and invasion assays in U87and LN229. (B) Representative image of TUNEL assay in U87 and LN229.

We validated the expression of these identified genes in glioma samples. The IHC staining results were retrieved from the HPA database. The IHC findings confirmed the increased expression of AEBP1, DCTD, DEPP1, DUSP6, FKBP9, and UGCG with the up‐regulation of glioma grades (Figure 8). We further explored the role of UGCG with two glioma cell lines. The expression of UGCG was tested at both transcriptomic and protein levels (Figure 9A). In U87 and LN229 cell lines, the knockdown of UGCG could decrease cell activity and proliferation in CCK8 and EdU assays (Figure 9B,C). Colony formation assays showed that the clonogenic ability was decreased (Figure 9D). Transwell migration and invasion assays were performed to evaluate cellular behavior, and the results showed a significantly decreased ability of migration and invasion of glioma cell lines after the knocking down of UGCG (Figure 10A). Moreover, we performed TUNEL assays to detect the apoptosis ability in glioma cell lines. It showed that the apoptosis ability was significantly increased (Figure 10B). Overexpression of UGCG could reverse these phenomena.

## 4 Discussion

Glioma accounts for about 80% of malignant brain tumors [32]. Improving the survival rate and accurately evaluating the prognosis are the major problems in glioma clinical management. Emerging therapeutic strategies seem to bring new hope for the prognosis of glioma patients, including targeted therapy, gene therapy, viral therapy, etc. [33, 34, 35]. Immunotherapy has shown efficacy across various tumor types, indicating potential applicability to different subtypes of glioma [36]. Immunotherapy could enhance the anti‐tumor response by modulating the activity of immune cells in the TME. Immunotherapy has the unique ability to induce immune memory, leading to ongoing surveillance and potential prevention of tumor recurrence. Furthermore, immunotherapy could be combined with other therapeutic strategies, such as radiotherapy, chemotherapy, and targeted therapy, to enhance efficacy through synergistic effects [37]. These indicate the great potential of immunotherapy in glioma treatment.

Neutrophils traditionally have been viewed as part of the innate immune system and primarily involved in the early stages of inflammation and defense against pathogens [38]. However, emerging evidence suggests that neutrophils can exhibit both tumor‐promoting and tumor‐inhibiting functions within the TME [39, 40]. The presence and phenotype of TANs have been correlated with clinical outcomes in various tumor types. Therefore, TANs can serve as prognostic biomarkers to predict patient response to immunotherapy and guide treatment decisions.

The efficacy of immunotherapy in glioma remains heterogeneous; addressing this challenge requires the identification of reliable biomarkers. The scRNA‐seq technologies enable the exploration of gene expression at the individual cell level, offering insight into the diverse cellular heterogeneity. In this study, we identified a distinct cluster characterized by elevated levels of neutrophil markers using a large sample of scRNA‐seq data. We then found a significant increase in neutrophil proportion in GBM compared to LGG, suggesting a potential role of these cells in tumor progression. These findings are consistent with the emerging evidence that neutrophil infiltration contributes to the aggressiveness of high‐grade gliomas. Furthermore, we identified specific DE‐NRGs and confirmed the biological functions of these genes. We then classified the glioma samples into molecular subtypes, representing a novel understanding of glioma heterogeneity. These molecular subtypes exhibited distinct clinical characteristics and prognoses, with C1 associated with worse outcomes. These findings underscore the importance of considering immune‐related factors in stratifying glioma patients, as they may have implications for prognosis and treatment selection. Moreover, immune‐related analyses revealed significant differences in the TME between molecular subtypes. We found that the C1 subtype exhibited higher immune and stromal scores, along with increased immune cell infiltration and immune checkpoint expression. Importantly, the elevated TIDE scores in the C1 subtype suggest a potential resistance to immunotherapy, highlighting the need for alternative treatment strategies in these patients. Functional enrichment analysis indicated the involvement of immune‐related pathways, emphasizing the relevance of immune modulation between these two subtypes.

Developing effective biomarkers represents a pressing research priority in glioma management. We developed the NRG‐related risk score model based on co‐expressed gene modules associated with distinct glioma subtypes. Central to this model are a set of hub genes, notably AEBP1, CAVIN1, DCTD, DEPP1, DUSP6, FKBP9, and UGCG, which exhibit considerable promise as prognostic indicators and potential therapeutic targets. AEBP1, for instance, has been implicated in promoting GBM progression through the activation of the NF‐κB pathway, with documented associations with enhanced cellular proliferation and survival in glioma cells [41]. Similarly, CAVIN1 has been linked to the invasive tendencies of GBM [42]. Studies have demonstrated the association between DCTD and worse prognoses in GBM patients undergoing concurrent capecitabine and radiotherapy [43]. A previous study showed that the knockdown of DEPP1 could significantly inhibit the growth of glioma cells both under hypoxia and normoxia [44]. Additionally, DUSP6 has shown significant up‐regulation and tumor‐promoting properties in GBM, while FKBP9 has been implicated in fostering the malignant behavior of GBM cells and conferring resistance to endoplasmic reticulum stress inducers [45, 46]. It has been shown that UGCG is related to neutrophil cells and immune response [47] and associated with the PI3K/AKT pathway [48], which is consistent with the functional enrichment analyses. UGCG has not been well studied in glioma. In this study, we performed in vitro experiments to investigate the role of UGCG within glioma cell lines. Our findings revealed that UGCG could enhance glioma cell proliferation, migration, and invasion while reducing apoptotic capacities.

The association of risk scores with clinical features further supports the clinical relevance of this model in guiding treatment decisions and prognostic assessment. The results of GSEA revealed enrichment of biological processes associated with neutrophil function and immune response, providing mechanistic insights into the role of neutrophils in glioma progression. These findings underscore the potential of targeting neutrophil‐related pathways as a therapeutic strategy in glioma. Additionally, the observed differences in mutation landscape between risk groups showed that the frequency of IDH mutation was significantly higher in the low‐risk group of both LGGs and GBMs. Drug sensitivity analyses indicated that the high‐risk group was less sensitive to TMZ and Vincristine, which might be a potential reason for the unfavorable outcomes in such patients. However, it was more sensitive to other common chemotherapeutic agents, highlighting the importance of personalized treatment approaches based on molecular subtypes and risk stratification. The NRG‐related risk score emerged as an independent prognostic factor for glioma patients, demonstrating robust predictive performance across multiple datasets. The construction of a nomogram incorporating prognostic features further enhances prognostic accuracy and clinical utility. The validation of prognostic value in external datasets underscores the generalizability and reliability of the risk score model, supporting its potential clinical application in guiding patient management. The validation of gene expression patterns through IHC staining further strengthens the findings of this study, providing evidence of the involvement of these selected genes in glioma progression. The consistent up‐regulation of gene expression with increasing glioma grade highlighted their potential as prognostic and therapeutic targets. Recently, it was shown that TANs and peripheral blood neutrophil levels before radiotherapy are prognostic of poor outcomes in GBM [49]. The results are consistent with our study. Additionally, we collected scRNA‐seq (51 glioma samples) and bulk RNA‐seq (2194 glioma samples) in the study and identified seven prognostic NRG‐related genes. There are many challenges faced by traditional single‐cell transcriptome sequencing. Immune cells, neurons, and tumor cells often exhibit significant heterogeneity, which could complicate the analysis. This diversity may lead to the presence of distinct cell states, making it challenging to identify and characterize specific populations of interest. scRNA‐seq provides high‐resolution data; the biological interpretation of results is a crucial challenge. Researchers also need to develop and apply suitable statistical models. Despite our efforts, this study still has several limitations. Firstly, it relied on retrospective analyses, underscoring the need for validation of the prognostic risk model in large‐scale, multi‐center prospective cohorts. Additionally, further investigation is warranted to elucidate the NRG‐related signaling pathways and regulatory mechanisms, enhancing our comprehension of their implications in glioma progression.

## 5 Conclusion

The study highlights significant disparities in both the proportion and functionality of neutrophils within the glioma TME. Furthermore, through the identification of distinct molecular subtypes based on DE‐NRGs, we have devised an NRG‐related risk score model. This model serves as a valuable prognostic tool and aids in stratifying treatment approaches for glioma patients. These findings enhance our understanding of glioma biology and carry significant implications for the advancement of novel immunotherapeutic strategies in glioma treatment.

## Author Contributions

Wen Wang and Junsheng Li contributed equally to this work and were considered co‐first authors. Wen Wang conducted all analyses. Junsheng Li interpreted the results and finalized the manuscript. Qiheng He and Chenglong Liu collected the data. Zhiyao Zheng and Siyu Wang created the figures and tables. Bojian Zhang, Siqi Mou, and Wei Sun revised the manuscript. Jizong Zhao conceived and designed the study. All authors have read and approved the final version of the manuscript. Wen Wang: Methodology, Validation, Software, Funding acquisition. Junsheng Li: Methodology, Validation, Writing‐original draft. Qiheng He: Data curation, Formal analysis. Chenglong Liu: Data curation, Formal analysis. Siyu Wang: Visualization. Zhiyao Zheng: Visualization. Bojian Zhang: Writing – review and editing. Siqi Mou: Writing review and editing. Wei Sun: Writing review and editing. Jizong Zhao: Methodology, Validation, Supervision, Project administration.

## Ethics Statement

This study was reviewed and approved by the Ethics Committee of Beijing Tiantan Hospital (KY2022‐178‐02).

## Consent

According to the publication guidelines of all public databases, informed consent was obtained from all participants.

## Conflicts of Interest

The authors declare no conflicts of interest.

## Supporting information


Data S1.

## Data Availability

The data that support the findings of this study are available from the corresponding author upon reasonable request.
