# Supplementary material for: Integrated Analysis to Reveal Heterogeneity of Tumor‐Associated Neutrophils in Glioma
Source: Cancer Med. 2025 Mar 7;14(5):e70745. doi: 10.1002/cam4.70745 (PMC11886415; doi:10.1002/cam4.70745)
Supplement: Supplementary file 1 — Data S1. [file CAM4-14-e70745-s001.docx]

**Supplementary materials**

**Table S1.** Primers for qRT-PCR.

| Gene | Sequence |
| --- | --- |
| UGCG-F | GCTCAGTACATTGCCGAAGAT |
| UGCG-R | AGCATTCTGAAATTGGCTCACA |
| GAPDH-F | ACGGATTTGGTCGTATTGGG |
| GAPDH-R | GGGATCTCGCTCCTGGAAG |

**Table S2.** Clinical characteristics comparison between different risk groups in TCGA database.

| Characteristics | Low-risk group | High-risk group | P value |
| --- | --- | --- | --- |
| Age, n (%) |  |  | <0.001* |
| ≤40 | 191 (54.9%) | 86 (24.8%) |  |
| >40 | 157 (45.1%) | 261 (75.2%) |  |
| Gender, n (%) |  |  | 0.852 |
| Female | 147 (42.2%) | 149 (42.9%) |  |
| Male | 201 (57.8%) | 198 (57.1%) |  |
| WHO grade, n (%) |  |  | <0.001* |
| G2 | 199 (57.3%) | 58 (16.7%) |  |
| G3 | 146 (42.1%) | 123 (35.4%) |  |
| G4 | 2 (0.6%) | 166 (47.8%) |  |
| IDH status, n (%) |  |  | <0.001* |
| Wildtype | 3 (0.9%) | 243 (71.9%) |  |
| Mutant | 344 (99.1%) | 95 (28.1%) |  |
| 1p19q status, n (%) |  |  | <0.001* |
| Non-codel | 196 (56.3%) | 323 (95.0%) |  |
| Codel | 152 (43.7%) | 17 (5.0%) |  |

*P<0.05, significant difference.

**Table S3.** Cox regression in TCGA database.

| Characteristics | Univariate analysis | | Multivariate analysis | |
| --- | --- | --- | --- | --- |
|  | Hazard ratio (95% CI) | P value | Hazard ratio (95% CI) | P value |
| Age |  |  |  |  |
| ≤40 | Reference |  | Reference |  |
| >40 | 4.252 (3.142-5.754) | <0.001* | 2.523 (1.755-3.627) | <0.001* |
| Gender |  |  |  |  |
| Female | Reference |  |  |  |
| Male | 1.246 (0.971-1.598) | 0.084 |  |  |
| WHO grade |  |  |  |  |
| G2 | Reference |  | Reference |  |
| G3 | 3.160 (2.174-4.592) | <0.001* | 1.918 (1.284-2.867) | 0.001* |
| G4 | 18.548 (12.662-27.171) | <0.001* | 4.409 (2.720-7.148) | <0.001* |
| IDH status |  |  |  |  |
| Wildtype | Reference |  | Reference |  |
| Mutant | 0.113 (0.086-0.147) | <0.001* | 0.499 (0.322-0.774) | 0.002* |
| 1p19q status |  |  |  |  |
| Non-codel | Reference |  | Reference |  |
| Codel | 0.226 (0.147-0.347) | <0.001* | 0.572 (0.348-0.940) | 0.028* |
| Risk score |  |  |  |  |
| Low | Reference |  | Reference |  |
| High | 6.287 (4.662-8.477) | <0.001* | 1.753 (1.121-2.742) | 0.014* |

*P<0.05, significant difference.

**Table S4.** Cox regression in CGGA-693 dataset.

| Characteristics | Univariate analysis | | Multivariate analysis | |
| --- | --- | --- | --- | --- |
|  | Hazard ratio (95% CI) | P value | Hazard ratio (95% CI) | P value |
| Age |  |  |  |  |
| ≤40 | Reference |  | Reference |  |
| >40 | 1.616 (1.292-2.022) | <0.001* | 1.344 (1.068-1.691) | 0.012* |
| Gender |  |  |  |  |
| Female | Reference |  |  |  |
| Male | 1.097 (0.882-1.363) | 0.405 |  |  |
| WHO grade |  |  |  |  |
| G2 | Reference |  | Reference |  |
| G3 | 2.899 (2.033-4.134) | <0.001* | 3.063 (2.143-4.376) | <0.001* |
| G4 | 7.783 (5.479-11.056) | <0.001* | 4.449 (3.052-6.485) | <0.001* |
| IDH status |  |  |  |  |
| Wildtype | Reference |  | Reference |  |
| Mutant | 0.326 (0.262-0.407) | <0.001* | 0.868 (0.642-1.173) | 0.357 |
| 1p19q status |  |  |  |  |
| Non-codel | Reference |  | Reference |  |
| Codel | 0.280 (0.199-0.393) | <0.001* | 0.520 (0.356-0.758) | <0.001* |
| Risk score |  |  |  |  |
| Low | Reference |  | Reference |  |
| High | 3.822 (3.034-4.814) | <0.001* | 2.078 (1.529-2.825) | <0.001* |

*P<0.05, significant difference.

**Table S5.** Cox regression in CGGA-325 dataset.

| Characteristics | Univariate analysis | | Multivariate analysis | |
| --- | --- | --- | --- | --- |
|  | Hazard ratio (95% CI) | P value | Hazard ratio (95% CI) | P value |
| Age |  |  |  |  |
| ≤40 | Reference |  | Reference |  |
| >40 | 1.627 (1.230-2.152) | <0.001* | 0.956 (0.707-1.293) | 0.772 |
| Gender |  |  |  |  |
| Female | Reference |  |  |  |
| Male | 0.935 (0.709-1.232) | 0.631 |  |  |
| WHO grade |  |  |  |  |
| G2 | Reference |  | Reference |  |
| G3 | 3.577 (2.339-5.471) | <0.001* | 3.165 (2.031-4.933) | <0.001* |
| G4 | 8.746 (5.886-12.996) | <0.001* | 5.262 (3.346-8.275) | <0.001* |
| IDH status |  |  |  |  |
| Wildtype | Reference |  | Reference |  |
| Mutant | 0.355 (0.268-0.470) | <0.001* | 1.325 (0.911-1.927) | 0.140 |
| 1p19q status |  |  |  |  |
| Non-codel | Reference |  | Reference |  |
| Codel | 0.170 (0.104-0.277) | <0.001* | 0.299 (0.174-0.512) | <0.001* |
| Risk score |  |  |  |  |
| Low | Reference |  | Reference |  |
| High | 4.578 (3.396-6.173) | <0.001* | 1.825 (1.211-2.750) | 0.004* |

*P<0.05, significant difference.

**Table S6.** Cox regression in REMBRANDT cohort.

| Characteristics | Univariate analysis | | Multivariate analysis | |
| --- | --- | --- | --- | --- |
|  | Hazard ratio (95% CI) | P value | Hazard ratio (95% CI) | P value |
| Age |  |  |  |  |
| ≤40 | Reference |  | Reference |  |
| >40 | 2.166 (1.232-3.806) | 0.007* | 1.157 (0.584-2.294) | 0.676 |
| Gender |  |  |  |  |
| Female | Reference |  |  |  |
| Male | 1.663 (0.991-2.789) | 0.054 |  |  |
| WHO grade |  |  |  |  |
| G2 | Reference |  | Reference |  |
| G3 | 2.190 (0.601-7.975) | 0.234 | 2.155 (0.558-8.314) | 0.265 |
| G4 | 9.406 (2.728-32.423) | <0.001* | 5.594 (1.498-20.893) | 0.010* |
| 1p19q status |  |  |  |  |
| Non-codel | Reference |  | Reference |  |
| Codel | 0.163 (0.058-0.453) | <0.001* | 0.285 (0.095-0.856) | 0.025* |
| Risk score |  |  |  |  |
| Low | Reference |  | Reference |  |
| High | 3.270 (1.990-5.373) | <0.001* | 2.096 (1.253-3.505) | 0.005* |

*P<0.05, significant difference.


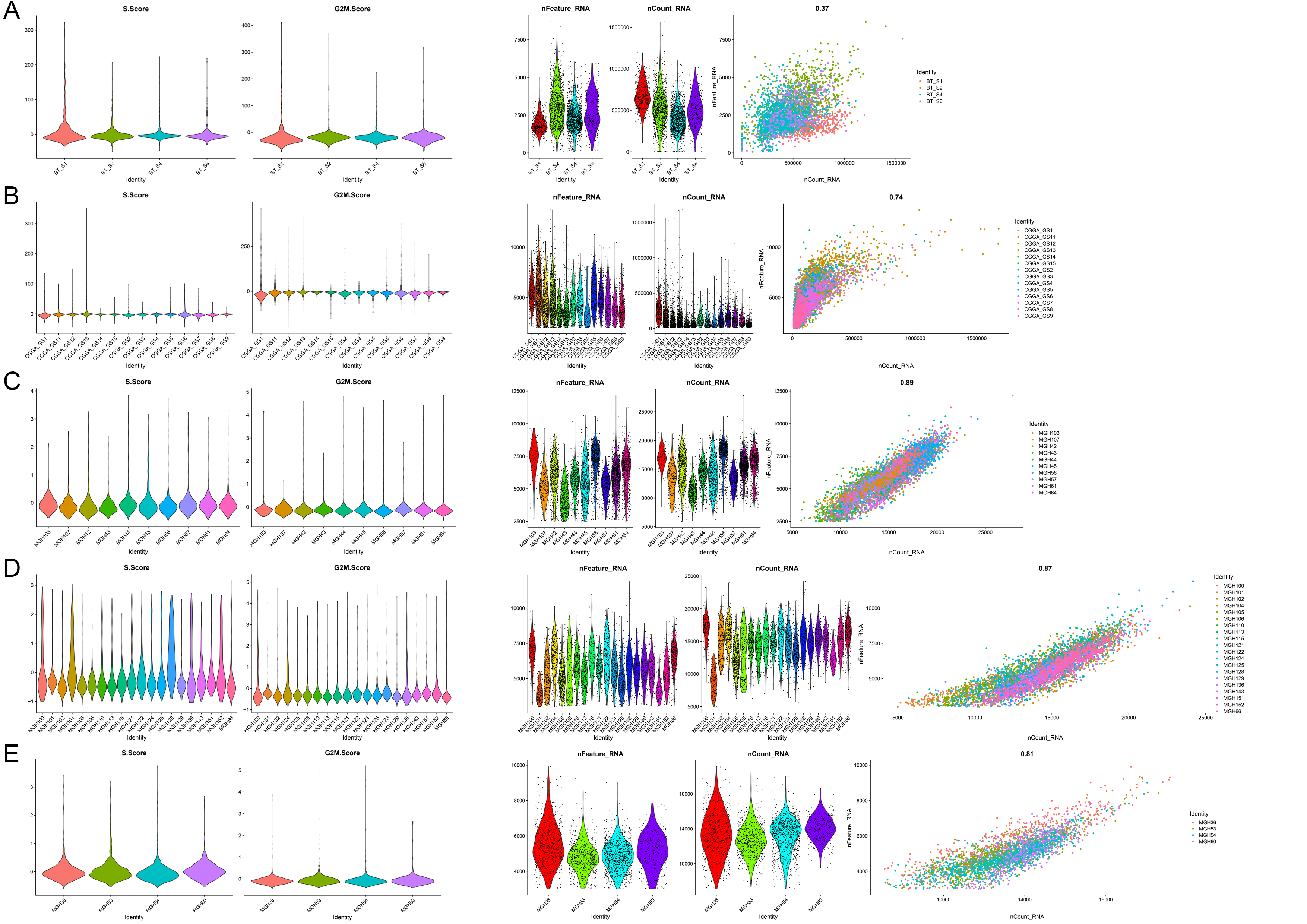


**Fig. S1.** Quality control and cell cycle evaluation for scRNA-seq.


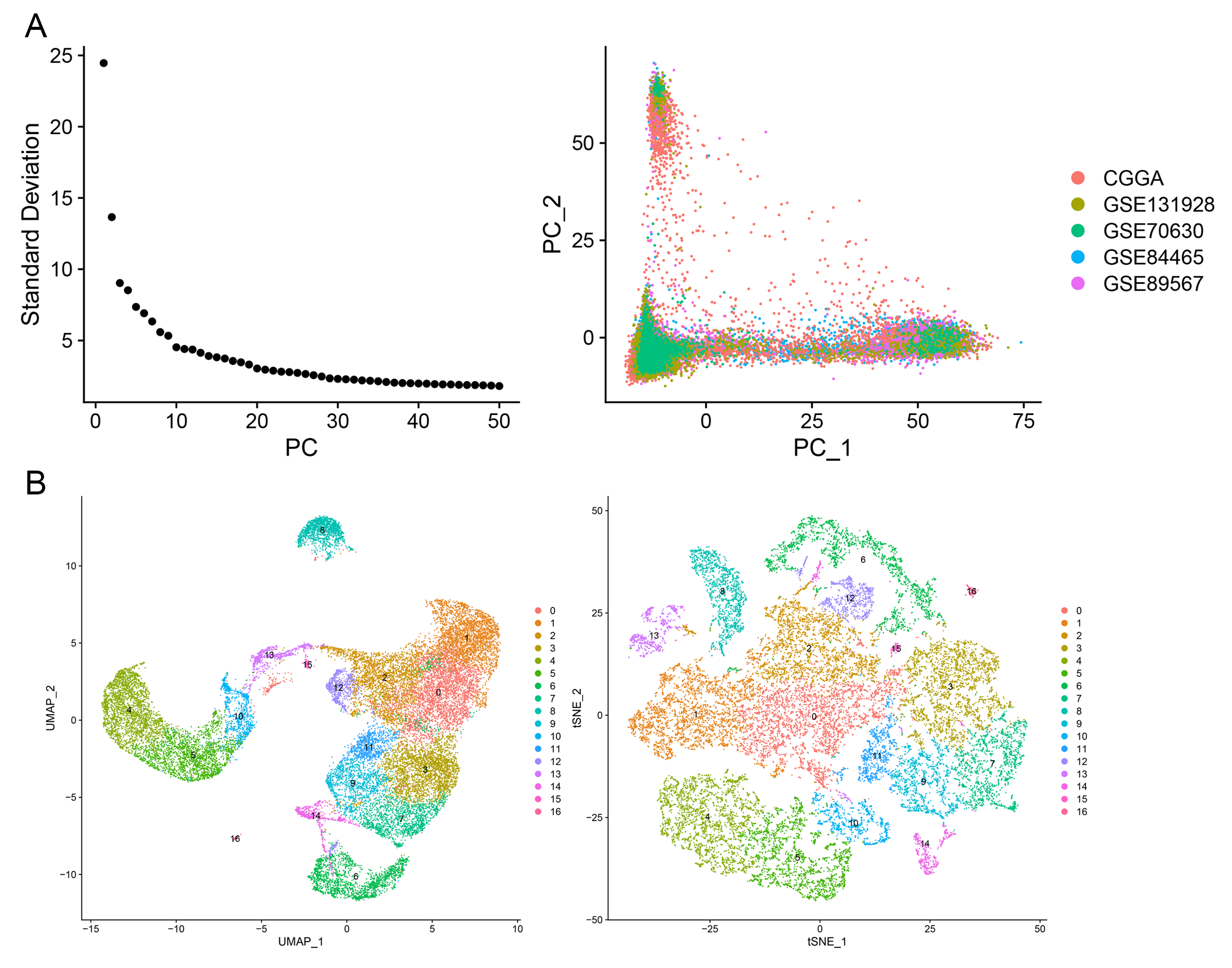


**Fig. S2.** Dimensionality reduction based on scRNA-seq data.

(A) Elbow plot and PCA plot.

(B) UMAP plot and tSNE plot.

**
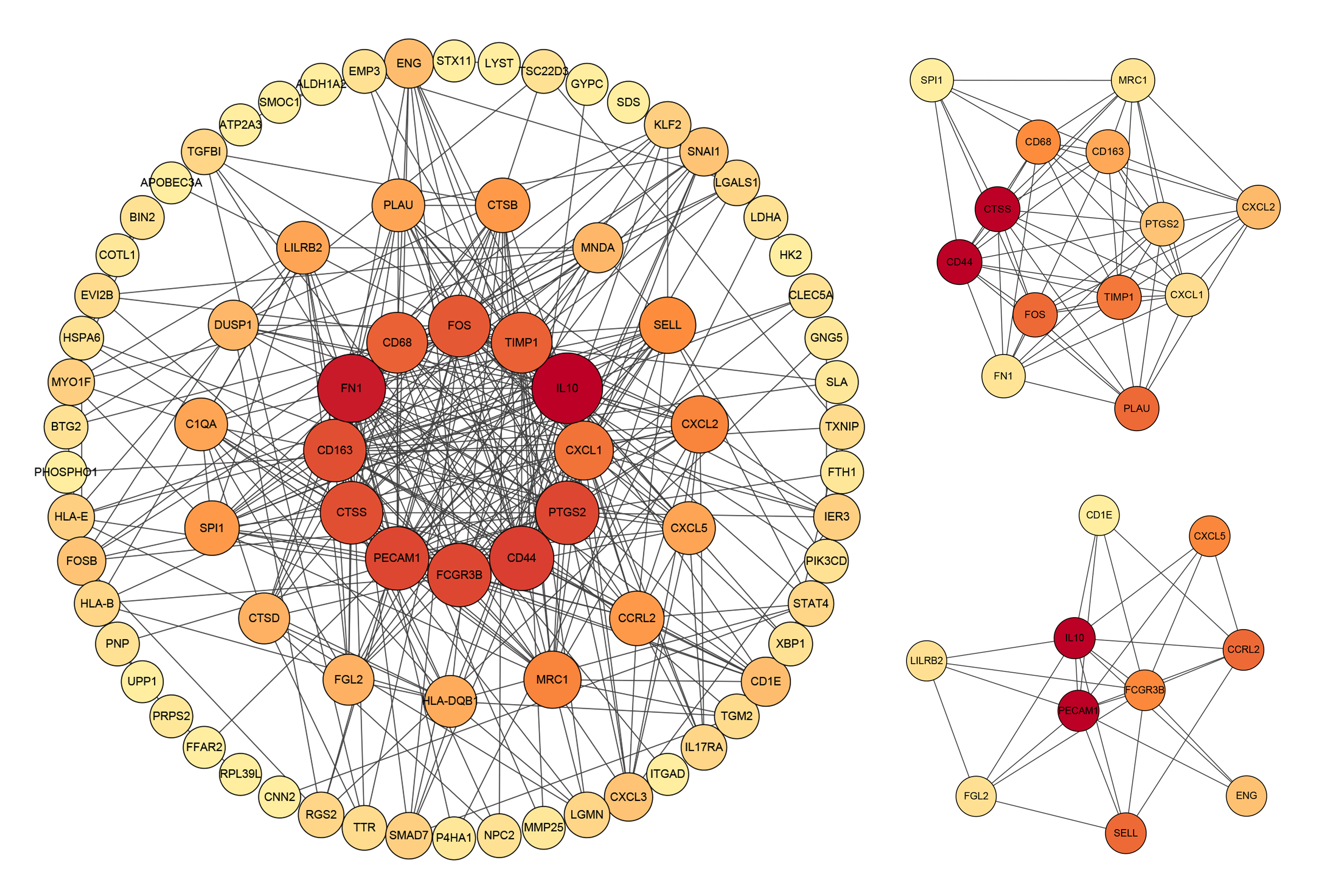
**

**Fig. S3.** Construction of PPI networks and identification of significant modules selected from PPI network.


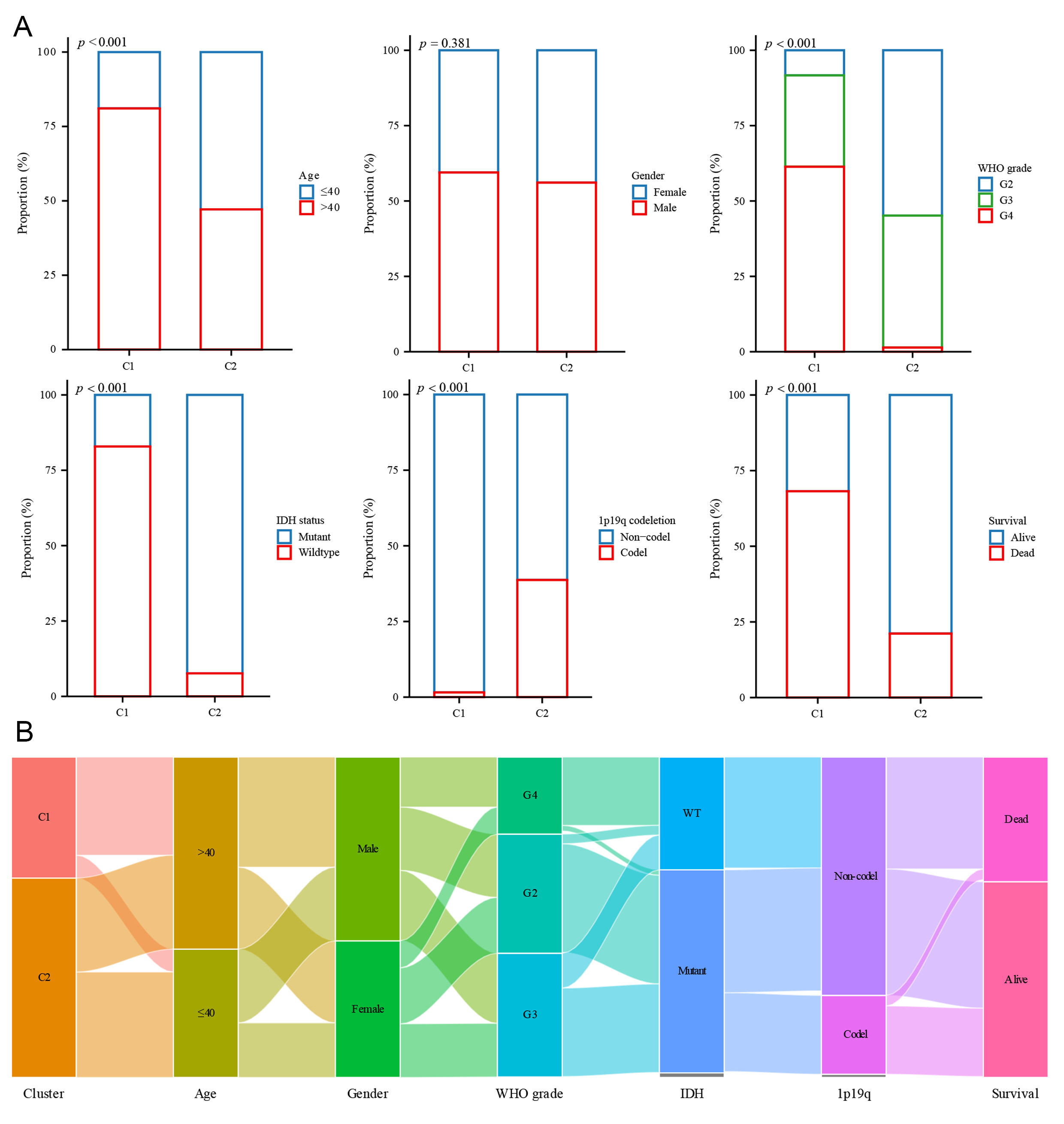


**Fig. S4.** Correlation between glioma subtypes and clinical characteristics.

(A) Distribution of clinical features between different subtypes.

(B) Sankey plot illustrating the relationship between clinical features and subtypes.


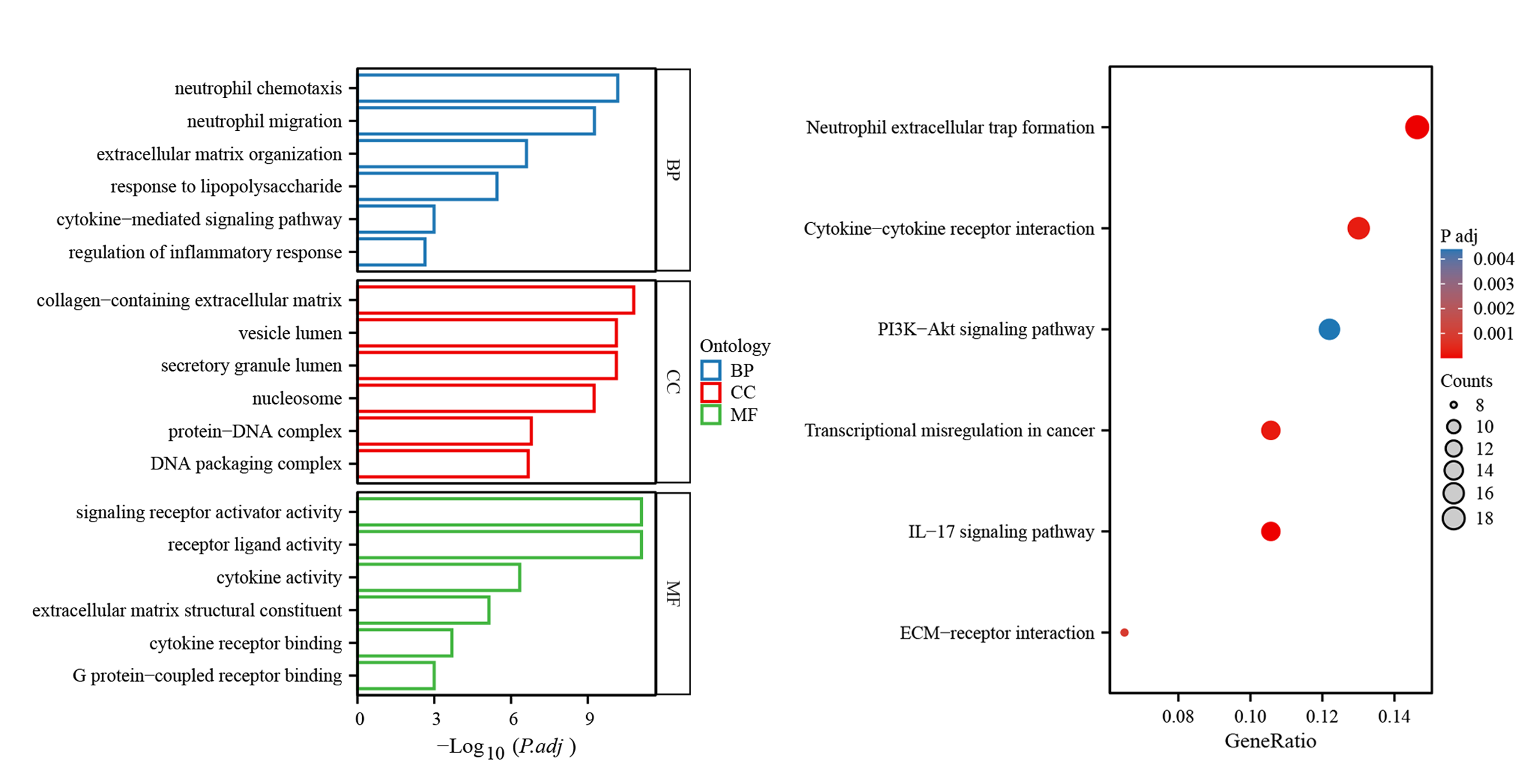


**Fig. S5.** Functional enrichment analyses between glioma subtypes, including GO and KEGG analyses.


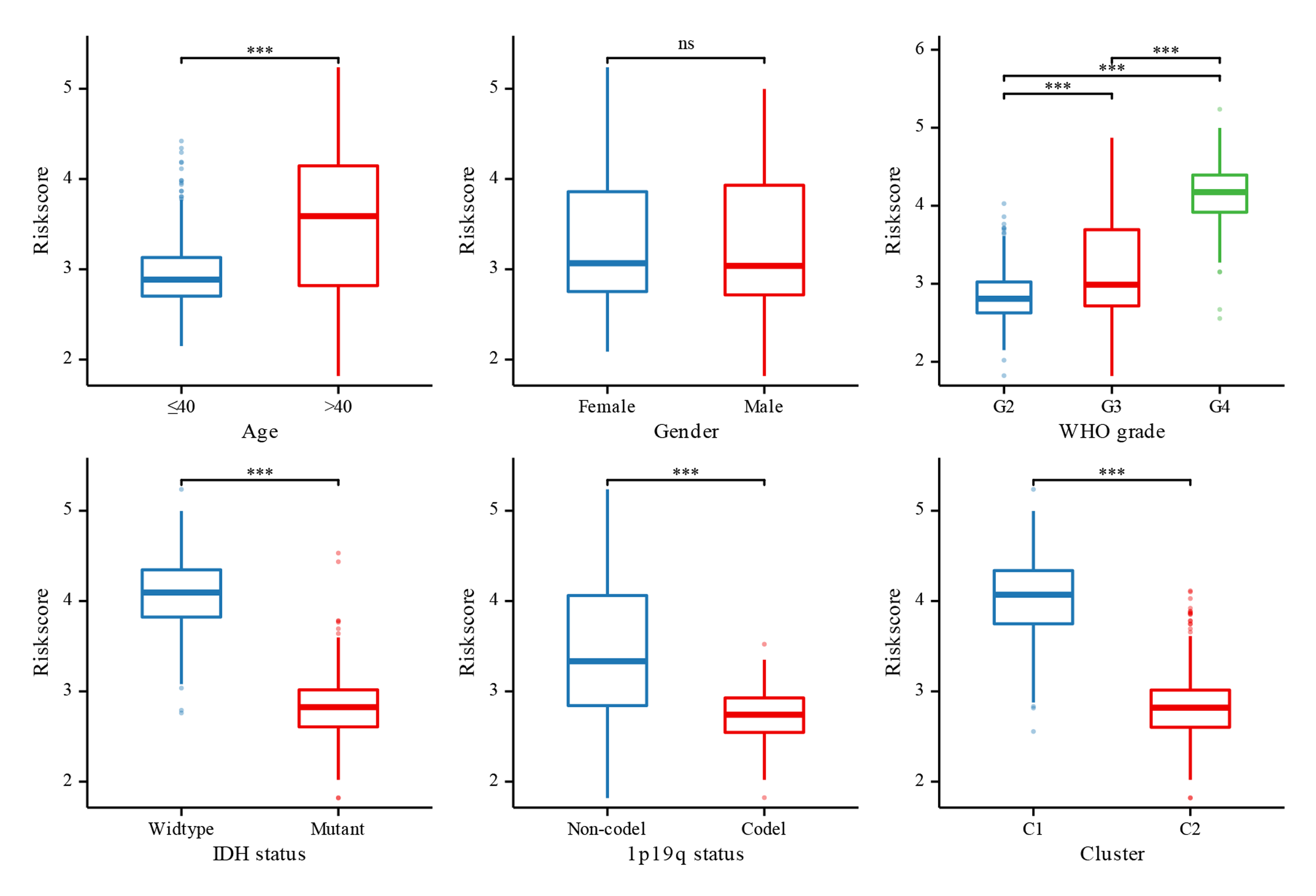


**Fig. S6.** Distribution of risk scores between different subgroups. ns, not significant; ***, P<0.001.


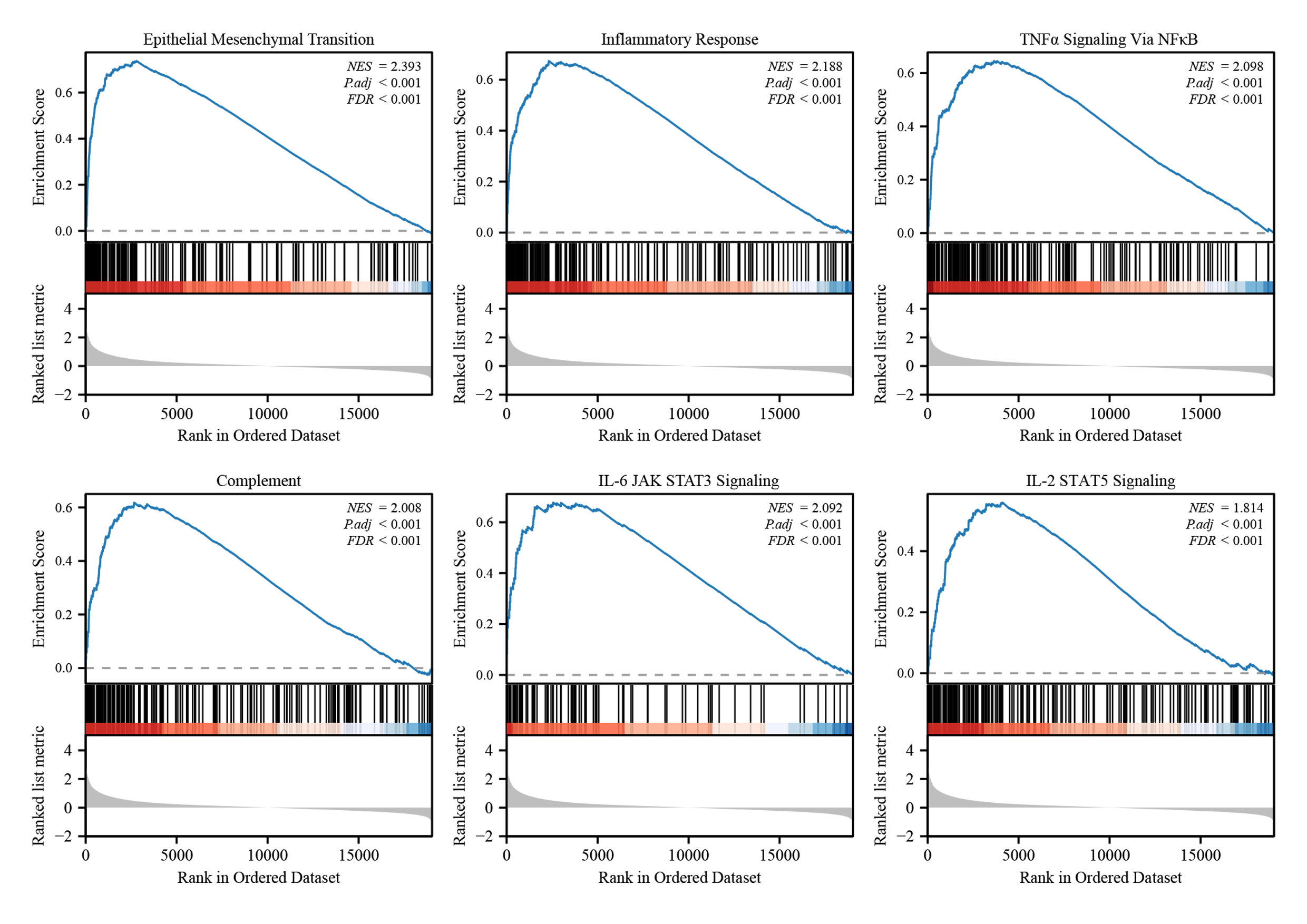


**Fig. S7.** GSEA analyses between low-risk and high-risk groups.


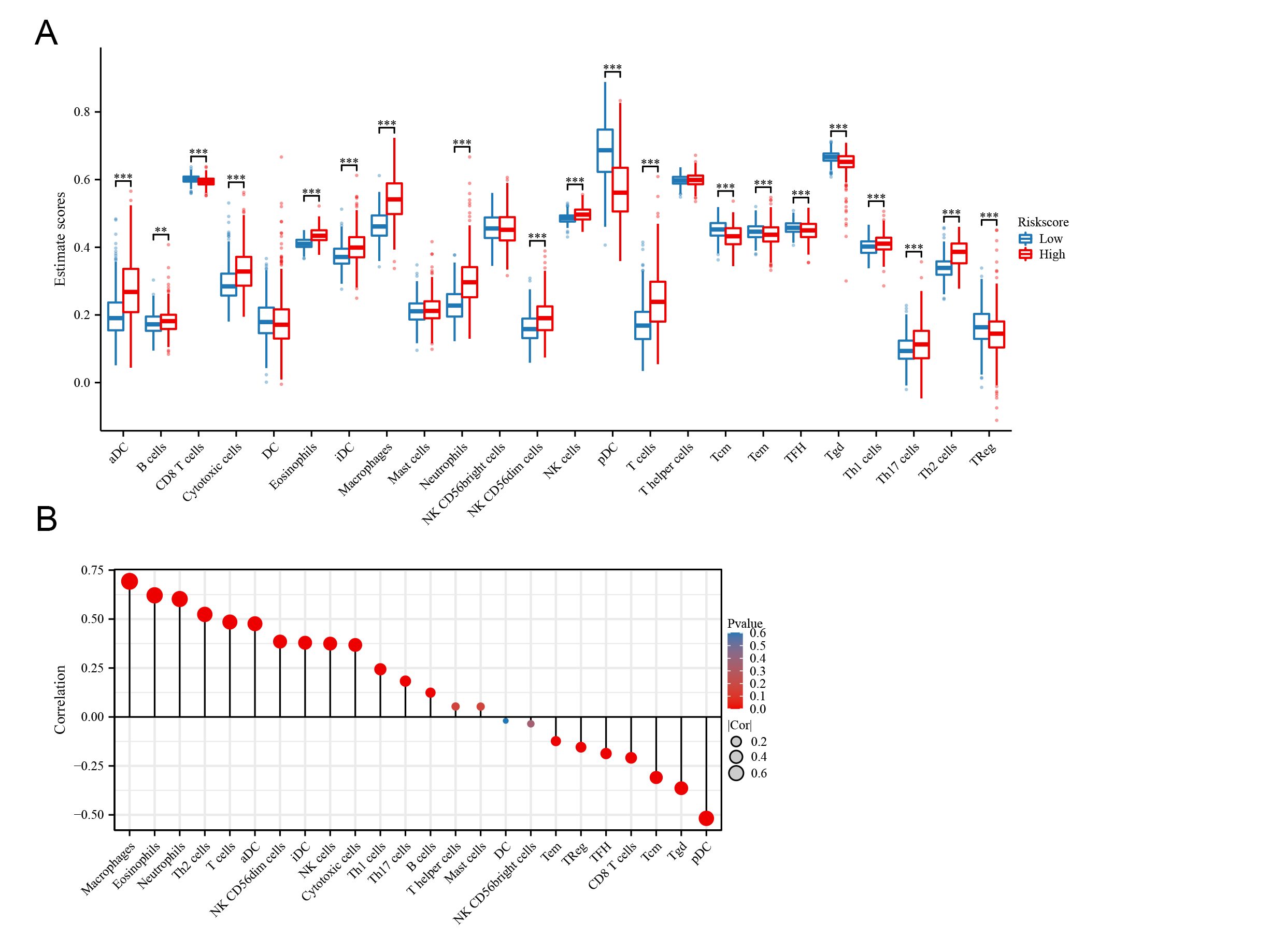


**Fig. S8.** Landscape of immune infiltration in different risk groups.

(A) Immune cell infiltration levels in different risk groups.

(B) Correlation between risk score and immune cell infiltration levels.


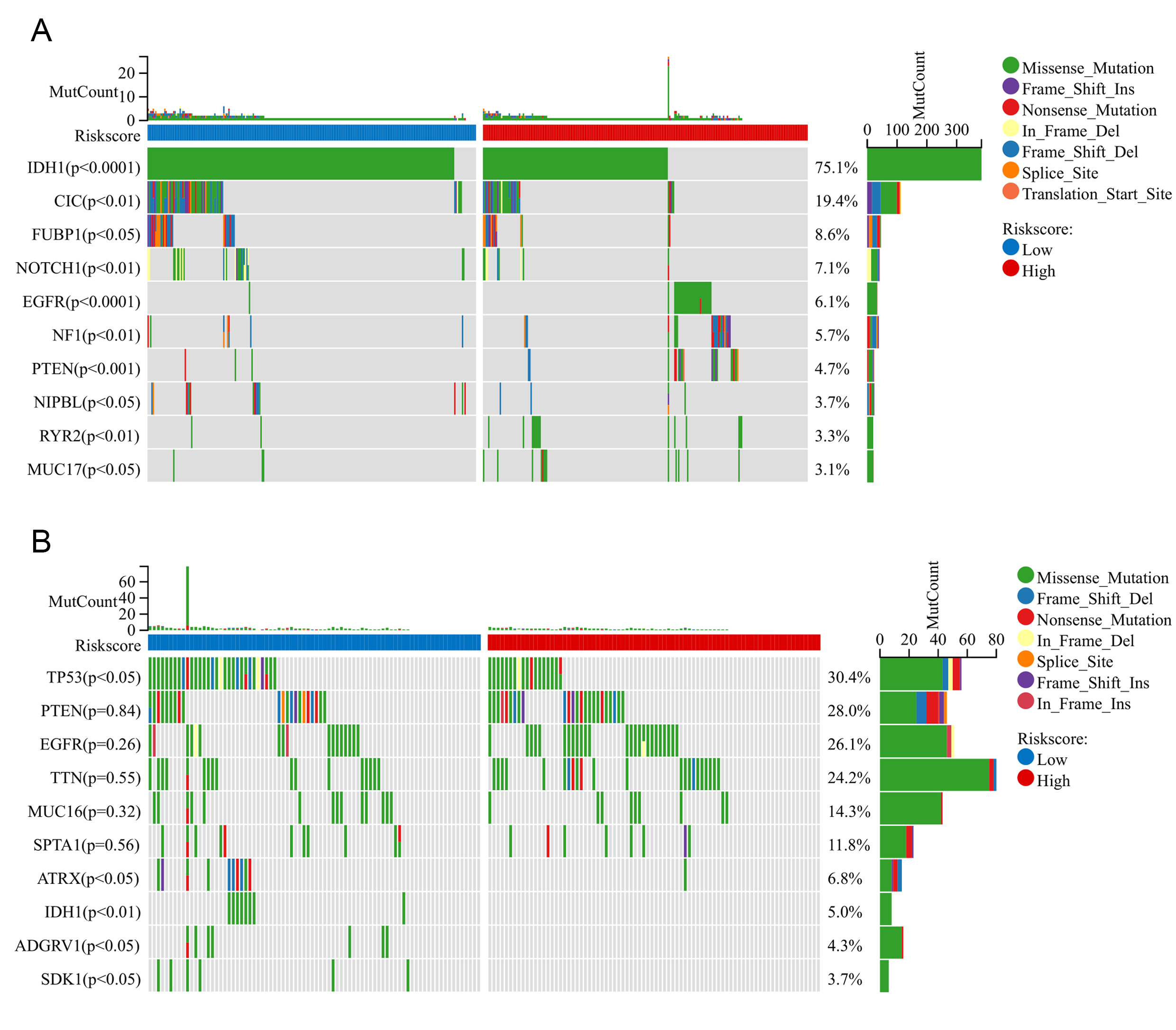


**Fig. S9.** Mutation landscape in different risk groups.


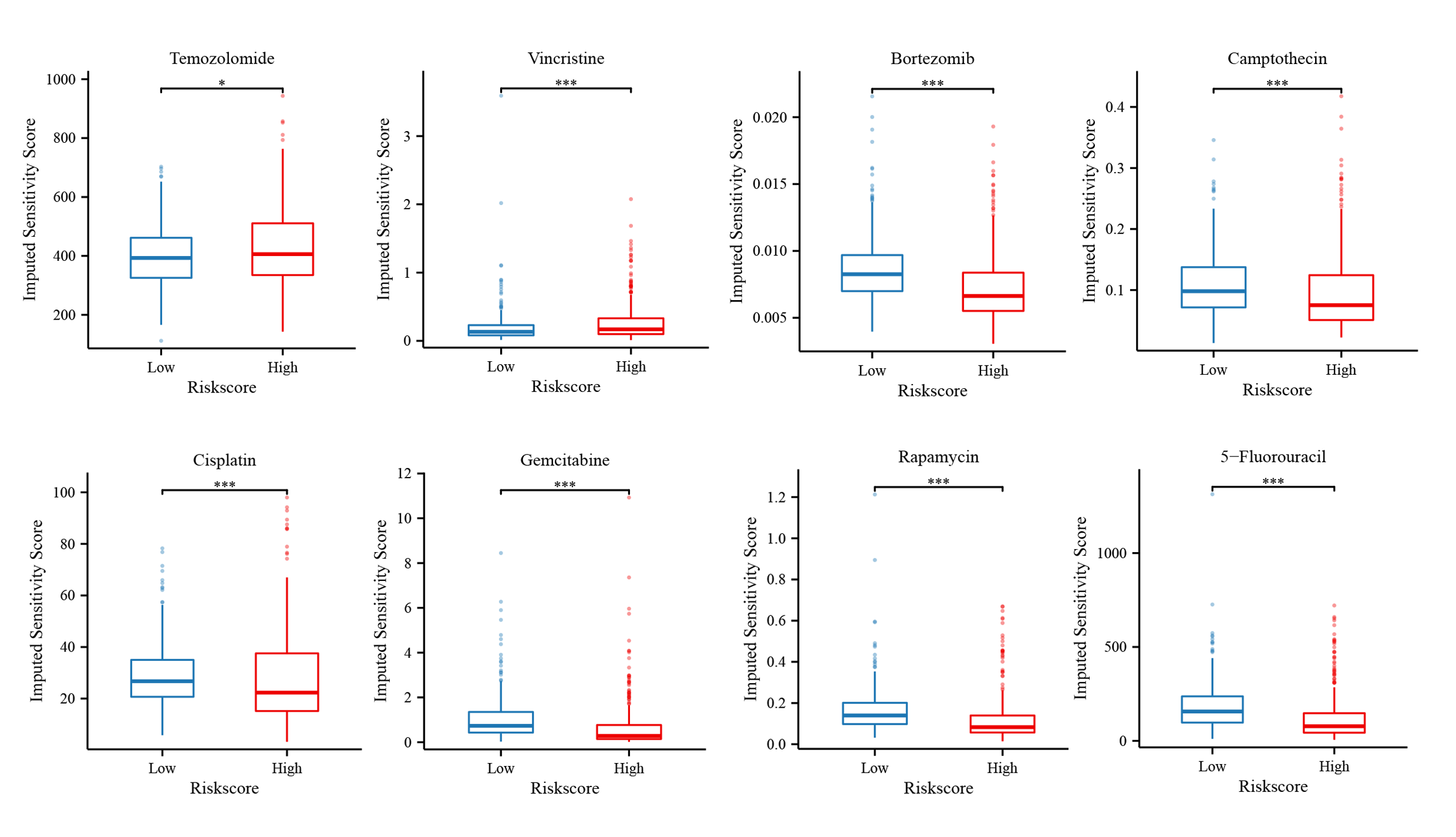


**Fig. S10.** Drug sensitivity to different chemotherapeutic agents in low-risk and high-risk groups.
